# Conversational Topic Shifts and Topic Maintenance in Autistic and Neurotypical Children

**DOI:** 10.1002/aur.70204

**Published:** 2026-02-18

**Authors:** Zuriñe Ábalos, Mikhail Kissine, Agustín Vicente, Elena Castroviejo

**Affiliations:** ^1^ University of the Basque Country (UPV/EHU) Vitoria‐Gasteiz Spain; ^2^ Université Libre de Bruxelles (ULB) Brussels Belgium; ^3^ University College London (UCL) London UK; ^4^ Ikerbasque: Basque Foundation for Science Bilbao Spain

**Keywords:** autism, children, communication, language, social interaction

## Abstract

Topic maintenance and topic shifts are crucial components of conversation; however, existing research lacks a clear quantitative operationalization of these topic management skills. Previous studies suggest that autistic children are less likely than their neurotypical peers to maintain and elaborate on the interlocutor's prior topic, and that they shift topics inappropriately more often. Nevertheless, findings on topic maintenance remain inconclusive, and studies specifically investigating topic shifts are limited. Moreover, little is known about the conversational skills of autistic children from non‐English‐speaking contexts. We investigated topic maintenance and shifting in 43 autistic and 46 age‐matched neurotypical Spanish‐speaking children (*M =* 8.55, SD *=* 1.91) during a semi‐spontaneous conversation task. Given their important role in social interactions, we developed a theoretically grounded protocol for systematically coding topic shifts, supported through a rating task conducted with neurotypical adults. Results showed that although autistic and neurotypical children provided a comparable number of topic‐supporting responses, autistic participants produced significantly more topic shifts. Furthermore, autistic children's topic shifts corresponded to a less natural end of the empirically supported rating scale, indicating such topic shifts interrupted the conversation flow more drastically. These findings suggest that, while autistic children may not have difficulties maintaining a conversation topic, the frequency and nature of their topic shifts could challenge reciprocal conversations. Our study presents a coding scheme that captures relevant distinctions in how different topic shifts are perceived in conversation, serving as a valuable resource for research and clinical practice in assessing and supporting the conversational skills of autistic individuals.

## Lay Summary

This study compared how autistic and neurotypical children maintained and shifted topics during conversation. Autistic and neurotypical children produced a similar number of on‐topic responses, but autistic children made more topic shifts, and their shifts were more drastic. These results suggest that while autistic children are able to maintain a conversation topic, their topic shifts can make their conversations harder to follow.

## 1 Introduction

Social communication and interaction difficulties are among the core diagnostic criteria for autism in the Diagnostic and Statistical Manual of Mental Disorders (DSM‐5). Central to these challenges are difficulties adhering to conversational norms, often resulting in what is described as “failure of normal back‐and‐forth conversation” (APA 2013).

The ability to maintain the interlocutor's previous topic is essential to engage in reciprocal conversations, which, in turn, is key for building and maintaining social relations (Friedman et al. 2019; Hazen and Black 1989). Autistic children's ability to maintain a conversation topic has been the focus of several studies, systematically reviewed in Ying Sng et al. (2018). Overall, autistic children are less *contingent* than neurotypical children, a contingent response being a response that contributes new or relevant information to develop the interlocutor's previous topic (Bloom et al. 1976; McGuinness et al. 2024; Nadig et al. 2010; Tager‐Flusberg and Anderson 1991). For instance, a contingent response to a question such as *Do you spend time with your family on holidays?* would be *Yes, and also with my friends*. Nevertheless, some studies also failed to find significant group differences in contingency (Abbot‐Smith et al. 2024). These discrepancies across studies may be due to methodological issues such as differences in coding schemes, participants' profiles or tasks (Ying Sng et al. 2018).

Non‐verbal (e.g., nodding) and minimal responses (e.g., *yeah*) have also been investigated as other types of topic‐supporting responses (Abbot‐Smith et al. 2024; see coding protocol in Abbot‐Smith et al. 2021). These types of responses, also referred to as forms of listener feedback (Matthewman et al. 2022), do not constitute elaboration on the previous topic, but instead serve as acknowledgments of the interlocutor's previous turn, showing interest and comprehension of the ongoing conversation. As such, these responses have been shown to positively correlate with conversation success (Matthewman et al. 2022), although an excessive reliance on them may hinder conversational flow (Abbot‐Smith et al. 2023). Abbot‐Smith et al. (2024) further subdivided minimal responses into *specific* to the content of the previous turn (e.g., *a doggy!; Have you?*) and *generic*, which could be produced without necessarily processing the preceding turn (e.g., *yeah; Mhm*). They found that autistic children were more likely than their neurotypical peers to provide generic minimal responses, while no group difference emerged for specific ones. Furthermore, both Matthewman et al. (2022) and Abbot‐Smith et al. (2024) reported that autistic children or adolescents were less likely to provide non‐verbal listener feedback.

Overall, autistic children seem to provide more non‐contingent responses than their neurotypical peers (Abbot‐Smith et al. 2024; McGuinness et al. 2024; Roberts et al. 2007). However, the definitions of non‐contingency vary across studies. For instance, Hale and Tager‐Flusberg (2005) define non‐contingent responses as utterances not related to the topic of the preceding utterance or utterances that are repetitions of what was previously said. Abbot‐Smith et al. (2024, 11) adopt a broader perspective: “Either (i) were not on the topic of (or only very tangentially related to) the immediately preceding turn, (ii) had a very unclear meaning or (iii) were ‘bizarre’ or (iv) switching to talk about something in the environment.” In Roberts et al. (2007), *don't know, yes* and *no* responses are also coded as non‐contingent when used to avoid continuing a topic.

In this study we focus on topic shifts (TS). There is a consensus that some TS are non‐contingent, but studies specifically investigating TS are scarce and often conflate TS with other variables (Ying Sng et al. 2018). For instance, Eales (1993) analyzed TS alongside underinformative utterances, and Bauminger‐Zviely et al. (2014) grouped them with several other pragmatic behaviors, such as irrelevant utterances and excessive talkativeness. Existing (limited) research indicates that autistic children produce more TS than their neurotypical peers (Bauminger‐Zviely et al. 2014; Loukusa et al. 2007; Paul et al. 2009; Roberts et al. 2007). These shifts are often described as “abrupt,” “unannounced,” “sudden,” “irrelevant,” or “proximally relevant” (Bauminger‐Zviely et al. 2014; De Villiers et al. 2007; Loukusa et al. 2007; Ochs and Solomon 2010; Paul et al. 2009). However, the lack of standardized definitions of TS makes it difficult to understand their nature and assess their impact on conversational coherence.

Importantly, different types of TS affect conversational flow in different ways, with some being frequent in naturally occurring discourse and even reflecting good conversational skills. Building on this, Roberts et al. (2007) distinguish between appropriate TS and inappropriate TS. Appropriate TS include an introducer (e.g., *by the way*) alerting the listener of a topic change, occur after a 3‐s pause or when the previous topic has been discussed at length, while inappropriate TS are abrupt, occur without pause time, or when the topic before has not been discussed at length. Roberts et al. (2007) found that neurotypical children produced a significantly higher proportion of appropriate TS than their autistic peers. While the distinction between appropriate and inappropriate TS as characterized represents an important advancement in the field, there remains room for further refinement. For instance, in terms of operationalization, it is not clear what qualifies as “discussing a previous topic at length.” Also, concerning the characterizations Roberts et al. (2007) offer, it is not explicitly stated whether a shift should meet all of the listed criteria, or whether satisfying one or two is sufficient to render it appropriate/inappropriate. Lastly, even meeting all the criteria does not ensure that a shift has been adequately taxonomized. For instance, in (1), the shift (in bold) exemplifies the presence of an introducer, a brief temporal gap and the resolution of the preceding topic. Yet, the shift does not seem to be appropriate:

1. A: Did you like yesterday's meal?
  B: Yes, I loved it, especially the lasagna… **By the way, dinosaurs are huge**.

One further reason for refining Roberts et al.'s classification is that TS are not necessarily categorical; rather, they can vary in their degree of pragmatic adequacy, as we will try to show.

The overarching goal of this study is to investigate the conversational topic management skills of Spanish‐speaking autistic and neurotypical children during a semi‐spontaneous conversation task. A key aim of our work is to develop a fine‐grained classification of TS, informed by rater perspectives and grounded in one of the most influential theories of discourse, which we can subsequently apply to analyze children's conversational data. This approach allows us to obtain nuanced data regarding the TS children produce, while identifying features of autistic children's TS that might be perceived as unusual or unnatural by neurotypical interlocutors, even in the absence of cognitive or language delays. Such an analysis is particularly relevant, as TS play a crucial role in navigating social interactions and shaping how children are perceived by their (autistic and neurotypical) peers (McGuinness et al. 2024; Place and Becker 1991). Moreover, most previous studies focus on English‐speaking children, and little is known about the conversational skills of autistic children from other linguistic or cultural contexts. Research across diverse cultural settings could help determine whether the observed differences between English‐speaking autistic and neurotypical children are widespread (Ying Sng et al. 2018). Our research questions are the following:

1. Do autistic and neurotypical children differ in the number of topic‐supporting responses they provide?
2. Do autistic and neurotypical children differ in the number of TS they produce? Are there differences in the nature of these TS that may affect conversational coherence differently?

Based on the diagnostic criteria for autism (APA 2013) and previous literature (Matthewman et al. 2022; McGuinness et al. 2024; Nadig et al. 2010), we predicted that, compared to their neurotypical peers, autistic children would provide fewer responses that maintain or elaborate on the previous topic. Moreover, we anticipated that autistic children would produce more TS and that their TS would have a greater impact on conversational coherence compared to those produced by neurotypical children (Bauminger‐Zviely et al. 2014; Loukusa et al. 2007; Paul et al. 2009; Roberts et al. 2007). In other words, we expected autistic children's TS to interrupt the conversation flow more drastically, potentially leading to negative perceptions in social interactions.

## 2 Methods

### 2.1 Participants

Forty‐three autistic and 46 neurotypical Spanish‐speaking 5–12‐year‐old children were included in this study. Diagnostic groups were matched on chronological age (*M =* 8.55, SD = 1.91), as confirmed by a Mann–Whitney *U*‐test (*W* = 1062, *p* = 0.55) (see Table 1 for descriptive statistics). Autistic participants possessed age‐appropriate receptive vocabulary skills, as assessed through the Spanish version of the PPVT‐III, Peabody: Test de Vocabulario en Imágenes (Dunn et al. 2010; IQ: *M* = 96.5, SD = 17.6; verbal mental age: *M* = 8.14, SD = 1.73). In addition, all autistic children had a non‐verbal IQ (NVIQ) score above 80 (*M* = 105, SD = 10.6), as measured by the Leiter‐3 scale (Roid et al. 2013).

**TABLE 1:** Participants' characteristics per group.

	Autistic	Neurotypical
*N* (M:F)	43 (38:5)	46 (22:24)
Mean age (SD)	8.67 (2.04)	8.43 (1.8)
Age‐range	5.6–12.11	6.5–12.1

Autistic children were recruited via the *Lindy Lab: Language in Neurodiversity Lab* at the University of the Basque Country (UPV/EHU) in Vitoria‐Gasteiz, the Biscayan autism association APNABI in Bilbao, and the *Matemáticas y Autismo Lab* at the University of Cantabria (UC) (Spain). Autistic participants had previously obtained a clinical diagnosis from a multidisciplinary assessment team. Two of these participants were also diagnosed with attention‐deficit hyperactivity disorder (ADHD). Neurotypical participants were recruited from a local school in Basauri (Basque Country, Spain), and parents confirmed that none of them had a history of psychiatric or neurodevelopmental diagnoses. Standardized tests could not be administered to neurotypical children due to time constraints within the school setting, which represents a limitation of this study. Nevertheless, since parents and schools were explicitly asked about any language or cognitive delays and none was reported, it is highly likely that the neurotypical group falls within the typical range for measures of receptive vocabulary and NVIQ. This study was approved by the Ethics Committee for research with human beings (CEISH) of the University of the Basque Country (UPV/EHU), code M10_2019_205. All parents signed the consent form for the treatment of the data and the participation of their children in the study.

### 2.2 Speech Data Collection

Each child engaged in a 20–30‐min semi‐spontaneous conversation with the experimenter. Conversations were elicited using the Eliciting Language Samples for Analysis (ELSA) (Barokova et al. 2021). ELSA is administered in a naturalistic play setting and consists of eight interactive activities (planting nuts, playing darts…) designed to maximize opportunities for eliciting language from the child. While the experimenter used prepared prompts for each activity, the conversations unfolded spontaneously. Conversations were video‐recorded and manually transcribed using the CHAT transcription format of the CHILDES project (MacWhinney 2000). Transcriptions and coding were performed by the first author using the CLAN software (MacWhinney 2000).

### 2.3 Coding

Building on Abbot‐Smith et al.'s (2021) coding protocol, we categorized children's responses to the experimenter's previous conversational turn into the following types: *contingent*, *minimal*, *topic shifts, missing* and *other*. Table 2 summarizes the coded response types; detailed coding instructions and examples of each coding category are provided in Supporting Information S1.

**TABLE 2:** Coding of response types.

Category	Definition
Contingent	Responses maintaining and elaborating on the immediate previous topic.
Minimal	Topic‐supporting (non‐)verbal responses without elaboration (e.g., *mhm, yeah*).
Topic shift	Responses deviating from the immediate previous topic.
Missing	When no (non‐)verbal response was provided after 2 s since the experimenter's turn, and the experimenter subsequently took another turn (see Pagmar et al. 2022).
Other	Responses that do not fit into any other category (e.g., clarification questions).

To code TS, we drew on the *Question Under Discussion* (QUD) discourse theory (Roberts 2012; Onea 2013, 2016; Riester 2019) and Van Kuppevelt's (1995) work. The QUD framework models discourse as a structured sequence of explicit and implicit questions that guide the flow of information, making it possible to identify when a contribution addresses the current question, introduces a new one or shifts away from it. This approach has been widely used in linguistics to annotate discourse and text (e.g., Riester et al. 2018). Based on notions developed within the QUD framework, we developed a novel coding scheme that distinguished three features of TS (for a theoretical discussion of these categories, see Ábalos et al., forthcoming):

- *Association*: refers to how closely the new topic (subject matter) relates to the preceding one. TS were coded as *associated* when they were thematically linked to the topic of the immediately preceding speech turn (i.e., the topic changed but still maintained a clear semantic connection to what had just been said), as *topic reintroductions* when linked to a non‐immediate previous topic, and as *non‐associated* when unrelated to prior discourse.
- *Marking*: captures whether the child overtly signaled the shift through a discourse marker such as *by the way* (*explicit*) or left it *implicit*.
- *Prompt*: accounts for the type of utterance (posed by the experimenter) immediately preceding the shift. TS were classified as following a *question*, a *statement*, or no prompt *(none)*. The latter included cases in which there was no utterance from the experimenter immediately preceding the shift, as well as instances where the experimenter's preceding utterance clearly closed the previous topic (e.g., through discourse markers such as *okay* or *great*). See Table 3 below for the definition and examples of each coding category:

**TABLE 3:** Coding of topic shifts.

Category	Subcategory	Definition	Example
Association	Associated	Related to the immediate previous topic.	A: I love cows. B: **(By the way,) they sleep 14 h**.
	Topic reintroduction	Related to a non‐immediate previous topic (i.e., one that has already been closed).	A: I love cows as well. Hey, when is your exam? B: Tomorrow. A: You will do great. B: **(By the way,) I also like dogs**.
	Non‐associated	Unrelated to any previous topic.	A: I love cows. B: **(By the way,) yesterday I went to the cinema**.
Marking	Explicit	A discourse marker (e.g., *by the way*) is used to signal the topic shift.	A: I love cows. B: **By the way, yesterday I went to the cinema**.
	Implicit	No discourse marker is used to signal the topic shift.	A: I love cows. B: **Yesterday I went to the cinema**.
Prompt	None	No question/statement from the experimenter immediately precedes the topic shift (the child first addresses the experimenter's previous topic and then subsequently introduces a new one), *or* the experimenter's preceding utterance clearly indicates the closure of the previous topic (e.g., *great, nice*).	A: I love cows. B: Me too. (**By the way,) yesterday I went to the cinema**. *or* A: Do you like cows? B: Yes, I do. A: Nice. B: (**By the way,) yesterday I went to the cinema**.
	Question	A question from the experimenter immediately precedes the topic shift.	A: Do you like cows? B: (**By the way,) yesterday I went to the cinema**.
	Statement	A statement from the experimenter immediately precedes the topic shift.	A: I love cows. B: (**By the way,) yesterday, I went to the cinema**.

*Note:* Topic shifts are highlighted in bold. The examples illustrating the categories “marking” and “prompt” involve non‐associated shifts (additional examples can be found in Supporting Information S1).

To illustrate how these features work, consider the following examples:

2 *Contingent response.*
  Experimenter: Do you like juice? What flavors do you like?
  Child: **Orange. That's the best flavor**.
3 *Associated topic shift.*
  Experimenter: Do you live in San Miguel?
  Child: Yes.
  Experimenter: I live here, near the town hall.
  Child: Ah. **Do you know how my mom refers to town halls?**
4 *Topic reintroduction.*
  Experimenter: What's your favorite animal?
  Child: Cats and monkeys. I saw monkeys at the zoo.
  Experimenter: Ah, how cool. Do you like dogs?
  Child: Mmm, yes, I like them.
  Experimenter: Ah, okay.
  Child: **You know what? Have you ever been to the zoo?**
5 *Non‐associated topic shift.*
  Experimenter: Who did you go hiking with?
  Child: With many teachers.
  Experimenter: Ah, with your school teachers? I thought you had gone with your family.
  Child: Yes. **Hey, look, I have a video game called “spy camera.”**

Although they may appear similar, contingent responses and associated TS are not equivalent. In a contingent response such as (2), the child directly addresses and elaborates on the topic raised by the experimenter. In (3), the conversation initially focuses on where each interlocutor lives, but the child subsequently shifts attention to a previously mentioned referent (i.e., the town hall), which becomes the new topic. Thus, while both types of responses maintain a connection to prior discourse, associated TS involve a change in conversational goal, whereas contingent responses do not. The shift in (3) would be specifically coded as associated, explicit (*do you know* functions as a discourse marker signaling the shift), no prompt (the child first responds to the experimenter's previous turn, closing the previous topic before shifting to the new one). The shift illustrated in (4) would be coded as topic reintroduction, explicit, no prompt (the previous topic has been closed by the experimenter uttering *ah, okay*). Finally, the shift in (5) would be non‐associated, explicit, no prompt.

To assess coding reliability, 10 randomly‐selected conversations (11% of the data) were double coded by a second independent coder, blind to the initial coding performed by the first coder (the first author). The agreement between the coders was excellent (Cohen's *κ* = 0.89). All disagreements were discussed and resolved.

### 2.4 Empirical Support for the Topic Shift Categories

The combination of the subcategories in our coding protocol (see Table 3) resulted in 18 different types of TS. In order to assess the impact of each type of shift on conversational coherence, we collected naturalness ratings from neurotypical adults. These ratings aimed to empirically support our theoretically‐informed coding scheme by confirming it captured relevant distinctions in how different TS are perceived in conversation, ultimately providing a robust tool for future research.

#### 2.4.1 Participants

A total of 100 neurotypical adults were recruited from the online platform Prolific. Participants were pre‐screened through Prolific's built‐in filters to exclude individuals with a history of neurodevelopmental conditions. Additionally, they had to meet the following inclusion criteria: (1) be between 18 and 55 years of age, (2) be native Spanish speakers and (3) be of Spanish nationality. After completing the experiment, four participants were excluded for failing one of the attention checks, resulting in a final sample of 96 participants (57 males, 38 females, 1 non‐binary). Participants' ages ranged from 21 to 55 years (*M* = 35.5, SD = 9.9).

#### 2.4.2 Procedure

Participants were presented with written transcripts of experimenter‐child conversations that took place during the ELSA task (see Section 2.2), all of which were fully anonymized. They were asked to rate the naturalness of the child's utterance in the context of the conversation using a seven‐point Likert scale, from 1 “not natural at all” to 7 “completely natural.” To build the stimuli, we carefully selected exchanges in which children produced clear TS. All exchanges were retrieved from conversations with an autistic child, as not all 18 types of TS were present in the neurotypical sample. However, participants were blind to the children's diagnosis, and, for that matter, that the study was related to autism. Furthermore, atypical features of the conversations (e.g., pedantic language) were removed to ensure that raters evaluated the child's utterance without being influenced by factors unrelated to conversation structure and TS. Additionally, the exchanges were standardized to consist of five conversational turns preceding the shift, with the final turn containing TS being the child's utterance participants were asked to evaluate. Additionally, exchanges without TS were also included as a baseline.

The experiment adopted a between‐subjects design, with participants divided into two lists (48 participants per list). Each list included all the 18 conditions, with participants rating two items per condition, resulting in 36 critical items per participant (across both lists, a total of 72 unique critical items were tested). Each participant also rated four baseline items, yielding a total of 40 items per participant. The items were presented in a randomized order. Additionally, two attention checks were included to ensure participant engagement (materials and instructions are provided in Supporting Information S2). The experiment lasted approximately 15 min and was programmed using PCIbex Farm (Zehr and Schwarz 2018). Due to the novelty of the experiment, it was first pretested with 26 participants. The pretest results showed a high internal consistency between the items, with a Cronbach's alpha of 0.93. Items receiving lower ratings compared to other items in the same condition were slightly modified before the main experiment to ensure that participants' judgments reflected the naturalness of the TS rather than confounds related to vocabulary, general knowledge or cultural familiarity (the items from the pretest are also included in Supporting Information S2).

## 3 Results

All statistical analyses were performed in R (R Core Team 2023). The complete R code, including model outputs and plots, can be found in the Supporting Informations. Results of the rating task are presented first, followed by the analysis of autistic and neurotypical children's conversations.

### 3.1 Naturalness Ratings of Topic Shifts

Ratings were analyzed with a cumulative link mixed model using the *clmm* function from the “ordinal” package (Christensen 2015), with condition included as fixed effect and participant and item as random effects. The *ggeffect* function from the “ggeffects” package (Lüdecke 2018) was used to extract the mean predicted ratings per condition, displayed in Table 4 below.

To assess which conditions differed significantly, post hoc analyses were carried out with the *emmeans* function from the “emmeans” package (Lenth et al. 2018). The baseline condition with no TS (condition 19 in Table 4) got significantly higher naturalness ratings than the rest of the conditions. Associated explicit TS with no prompt (condition 1) were the only exception, and did not differ from the baseline (*p* = 0.10). These were explicitly marked TS that were thematically related to the previous topic and that occurred after acknowledging the experimenter's previous turn (see example (3) above). Additionally, most of the non‐associated TS (conditions 14–18) received significantly lower ratings than other conditions, with non‐associated explicit TS with no prompt (condition 13)—illustrated in example (5)—overlapping more with topic reintroductions and associated TS (these results are shown in Supporting Information S2). Since pairwise comparisons alone made it difficult to group conditions together, a cluster analysis was performed to explore potential groupings among the conditions based on their rating patterns, providing a clearer understanding of how the different TS might be related to each other in terms of perceived naturalness.

K‐means clustering was applied with the *kmeans* function to the conditions based on their mean predicted ratings obtained through the cumulative link mixed model. The Elbow method determined the optimal number of clusters was three (see Table 4 below). A one‐way analysis of variance performed with the *anova* function revealed significant differences between the clusters (*F*(2, 16) = 81.98, *p* < 0.001).

**Cluster 1. Least natural**. Conditions with the lowest ratings were grouped in cluster 1, including all non‐associated TS (i.e., those thematically unrelated to any prior topic) except for explicit ones produced after having addressed the prior topic (non‐associated explicit TS with no prompt; example (5)).

**Cluster 2. Intermediate naturalness**. Cluster 2 was composed of non‐associated explicit TS with no prompt (example (5) above) and all topic reintroductions with a single exception: explicit topic reintroductions with no prompt (example (4) above). Cluster 2 also contained a subset of associated TS: those following the experimenter's question (e.g., Experimenter: *Let's get to a place…* Child: *There're animals!* Experimenter: *You really liked animals, didn't you?* Child: ***This cow has a wound***) and explicit ones following the experimenter's statement (e.g., Experimenter: *I rode a horse once. The horse started running. I never rode one again*. Child: ***Well, there are also white horses***).

**Cluster 3. Most natural**. Cluster 3 contained the remaining associated TS, together with explicit topic reintroductions with no prompt, illustrated in example (4).

**TABLE 4:** Mean predicted ratings, standard deviations (SD) and cluster assignment per topic shift type (condition).

Condition	Association	Marking	Prompt	Mean rating (SD)	Cluster
1	Associated	Explicit	None	5.94 (1.17)	3
2	Associated	Explicit	Question	4.42 (0.44)	2
3	Associated	Explicit	Statement	4.82 (0.57)	2
4	Associated	Implicit	None	5.61 (0.93)	3
5	Associated	Implicit	Question	4.18 (0.37)	2
6	Associated	Implicit	Statement	5.44 (0.83)	3
7	Topic reintroduction	Explicit	None	5.20 (0.71)	3
8	Topic reintroduction	Explicit	Question	4.17 (0.37)	2
9	Topic reintroduction	Explicit	Statement	4.81 (0.56)	2
10	Topic reintroduction	Implicit	None	4.69 (0.52)	2
11	Topic reintroduction	Implicit	Question	4.19 (0.38)	2
12	Topic reintroduction	Implicit	Statement	4.49 (0.46)	2
13	Non‐associated	Explicit	None	4.08 (0.35)	2
14	Non‐associated	Explicit	Question	2.89 (0.14)	1
15	Non‐associated	Explicit	Statement	2.22 (0.14)	1
16	Non‐associated	Implicit	None	2.77 (0.13)	1
17	Non‐associated	Implicit	Question	1.74 (0.20)	1
18	Non‐associated	Implicit	Statement	2.29 (0.13)	1
19	Baseline	Baseline	Baseline	6.69 (2.08)	3

*Note:* The baseline condition included no topic shifts. Ratings ranged from 1 (“not natural at all”) to 7 (“completely natural”). Cluster 1 grouped the conditions with the lowest ratings, while cluster 3 included those with the highest ratings.

### 3.2 Comparison Between Autistic and Neurotypical Children's Topic Management Skills

Children's mean length of utterance (MLU) was extracted as a proxy for language level (see, for instance, Tager‐Flusberg and Anderson 1991). A linear regression, with group as fixed effect and age (in months) and number of conversational turns as control variables, revealed no significant differences between autistic and neurotypical children (*p* = 0.34). Descriptive statistics of conversational turns, MLU and response types per group are presented in Table 5 below. Notably, while most conversations with autistic children lasted 30 min, those with neurotypical children had an average duration of 22 min, due to logistic constraints.

**TABLE 5:** Descriptive statistics of conversational turns, mean length of utterance (MLU) and response types per group.

	Autistic			Neurotypical		
	Total	Mean (SD)	Percentage	Total	Mean (SD)	Percentage
Turns	10,610	247 (42.6)	NA	8496	185 (27.1)	NA
MLU	NA	3.46 (0.85)	NA	NA	3.34 (0.97)	NA
Contingent	5219	121 (30.9)	49%	4553	99 (19.9)	54%
Minimal	3173	73.8 (28.7)	30%	3291	71.5 (22.1)	39%
Topic shift	483	11.2 (9.64)	5%	112	2.43 (3.62)	1%
Missing	901	21 (14.1)	8%	294	6.39 (6.57)	3%
Other	834	19.4 (13.7)	8%	246	5.35 (4.48)	3%

*Note:* To offer a more standardized representation of the data, percentages were calculated by dividing the total number of each response type by the total number of conversational turns.

**FIGURE 1:**
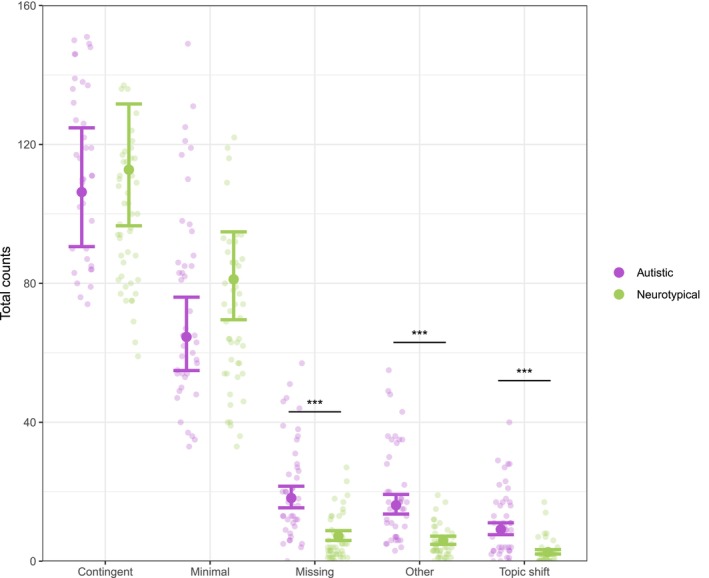
Total counts of response types per participant and group with fitted values and 95% confidence intervals.

To model counts of response types, a mixed‐effects negative binomial regression was fitted using the *glmer.nb* function from the “lme4” package (Bates et al. 2015), with counts as the dependent variable, and group, response type and their interaction as fixed effects. By‐participant intercepts were included as a random effect. Additionally, age (in months) and number of conversational turns were controlled for by including them as fixed effects. The model revealed significant group‐response type interactions, displayed in Figure 1. Post hoc pairwise comparisons, using the *emmeans* function, showed that autistic children produced significantly more topic shifts (*β* = 1.26, SE = 0.16, *z* = 7.97, *p* ≤ 0.001), missing (*β* = 0.92, SE = 0.14, *z* = 6.78, *p* ≤ 0.001) and other (*β* = 1.01, SE = 0.14, *z* = 7.25, *p* ≤ 0.001) responses. No significant differences were detected for contingent and minimal responses (both *p* ≥ 0.05).

**FIGURE 2:**
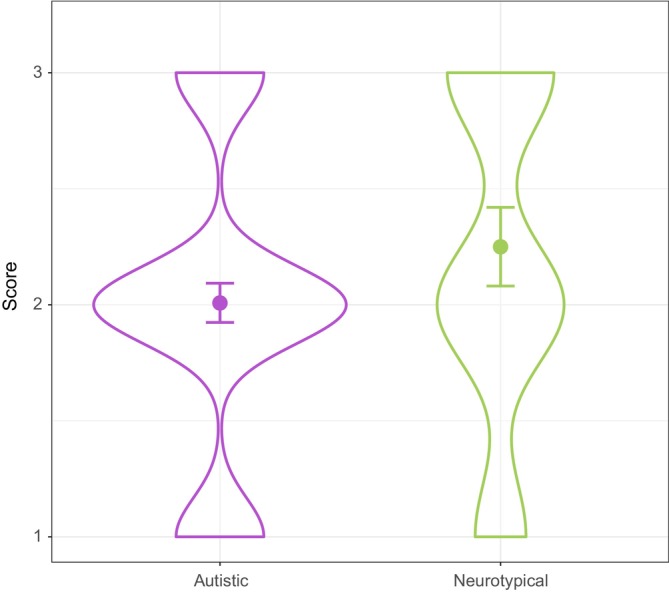
Distribution of topic shift naturalness scores per group with fitted values and 95% confidence intervals.

To examine the types of TS each group produced (see Table 6 below for the total counts and percentages of TS per cluster and group), each shift was assigned a naturalness score, from 1 to 3, based on the cluster to which it belonged (see Table 4 above). In this scoring scheme, 1 corresponded to the least natural type of shifts (cluster 1), 2 to shifts of intermediate naturalness (cluster 2), and 3 to the most natural TS (cluster 3). A linear mixed‐effects regression was performed using the *lmer* function from the “lme4” package (Bates et al. 2015), with scores as a dependent variable, group as a fixed effect, participant as a random effect, and age and number of conversational turns as control variables. The model revealed a significant group effect, with the autistic group obtaining significantly lower scores than their neurotypical counterparts (*β* = 0.24, SE = 0.10, *t* = 2.45, *p* = 0.02); see Figure 2.

**TABLE 6:** Total counts and percentages of topic shifts per cluster and group.

Cluster	Autistic		Neurotypical	
	Total	Percentage	Total	Percentage
1	99	20.5%	20	17.9%
2	285	59%	50	44.6%
3	99	20.5%	42	37.5%

*Note:* Score 1 was assigned to the topic shifts receiving the lowest naturalness ratings, while score 3 was assigned to those rated as the most natural. Percentages were calculated by dividing the number of topic shifts receiving each score (1, 2, or 3) by the total number of topic shifts.

## 4 Discussion

The present study is, to our knowledge, the first to investigate the differences between the topic management skills of Spanish‐speaking autistic and neurotypical children. Given the crucial role TS play in social interaction, we aimed at providing a fine‐grained analysis of the ways in which these children drifted from the previous topic, while also contributing to the existing body of research on their topic maintenance skills.

### 4.1 Topic Maintenance

Concerning our first research question, autistic children did not significantly differ from their neurotypical peers in their ability to maintain the conversation topic. Both groups of children produced a comparable number of contingent responses (see Figure 1), meaning that they were equally likely to elaborate on the interlocutor's previous topic, in line with Abbot‐Smith et al.'s (2024) results. However, this finding contradicts previous research showing a reduced level of contingency in autistic children (McGuinness et al. 2024; Nadig et al. 2010). A possible explanation may be that we used ELSA to elicit conversation samples. ELSA is designed to promote naturalistic conversation within a joint activity framework, allowing for a dynamic exchange between the child and the experimenter. Engaging in a shared activity can offer a structured yet flexible framework that reduces the cognitive and communicative demands associated with maintaining a conversation. Moreover, although the experimenter used certain prompts when administering the ELSA task, the conversation topics varied based on the child's interests, while studies coding children's responses to conversation probes often involve specific, predetermined topics (Abbot‐Smith et al. 2024; McGuinness et al. 2024). Although those topics may be of interest to the child, the production of contingent responses could be affected if the subject matter does not align with the child's preferences, as conversational topics may play a role in social responsiveness (Clin et al. 2024). Thus, we believe that the nature of the ELSA task may have helped autistic children showcase their strengths in maintaining engagement in conversation, making it a valuable tool for assessing their conversational skills. Furthermore, children appeared to thoroughly enjoy the activity, further highlighting its effectiveness.

Moreover, although approaching significance, no significant group differences were found regarding the number of minimal responses provided (see Figure 1). These responses could be interpreted as instances of listener feedback, which autistic individuals have been shown to provide less frequently. However, previous studies identified group differences only in certain forms of listener feedback, specifically in non‐verbal responses (Abbot‐Smith et al. 2024; Matthewman et al. 2022). In addition, Abbot‐Smith et al. (2024) found that autistic children were more likely than their neurotypical counterparts to produce *generic* minimal responses (e.g., *mhm*). The fact that we did not distinguish between different types of minimal responses (since this was not a primary aim of the study) may have led us to overlook differences reported in previous studies.

### 4.2 Topic Shifting

#### 4.2.1 Naturalness Ratings of Topic Shifts

A central aim of this study was to gain a fine‐grained understanding of whether and how the types of TS produced by autistic and neurotypical children differed, with a particular focus on identifying those patterns that might be perceived as unnatural by neurotypical interlocutors. To this end, we developed a theory‐informed coding scheme which could be subsequently used in profiling communicational difficulties and targeting interventions.

Our results, based on the naturalness ratings provided by neurotypical adults, provided empirical support for the theoretical distinction we proposed. As expected, the baseline condition without TS was rated the highest in naturalness (see Table 4), supporting the importance of topic maintenance for effective conversations. In contrast, thematically unrelated (i.e., non‐associated) TS were rated as the least natural. Notably, however, non‐associated TS that were explicitly marked and produced after having addressed the previous topic (example (5)) received higher ratings, indicating that some form of connection or acknowledgment of the prior topic may mitigate perceived disruption. Topic reintroductions were rated as moderately natural, likely due to their connection to earlier parts of the conversation, but not entirely natural, as they involved reopening a topic that had already been closed. Explicit topic reintroductions following an acknowledgment of the prior turn (example (4)) were rated higher than other types of topic reintroductions, mirroring non‐associated TS. Finally, associated TS were perceived as the most natural TS, except when occurring after the interlocutor's question, which were rated lower (note that shifting topic implies leaving the question unanswered). In a nutshell, this rating task provided empirical ground to our suspicion that different types of TS affect conversational flow in different ways. The three coded dimensions, among which association appears to be the most influential, interact to determine the naturalness of a shift. Notably, the association dimension was not included in previous coding schemes, such as Roberts et al.'s (2007). To our knowledge, no previous study has systematically investigated how specific linguistic features of TS influence their perceived naturalness. Accordingly, the proposed categories remain open to replication and refinement in future work.

#### 4.2.2 Topic Shifts in Autistic and Neurotypical Children

Regarding our second research question, although autistic children demonstrated an ability to maintain and elaborate on the previous topic, the results showed that they deviated from this topic significantly more often than their neurotypical peers, a finding which is consistent with previous literature (Bauminger‐Zviely et al. 2014; Loukusa et al. 2007; Paul et al. 2009; Roberts et al. 2007). These results raise the possibility that when neurotypical children are not interested in or do not fully grasp the conversational topic—an ability that is not always straightforward to master (Abbot‐Smith et al. 2023)—they may be more inclined to give minimal responses. In contrast, autistic children may be more likely to shift topic (or provide no answer, as suggested by the significantly higher number of missing responses). This interpretation remains speculative and requires further investigation.

In addition, findings revealed significant group differences in the types of TS children produced. Specifically, autistic children's TS corresponded to a less natural end of the empirically supported scale (i.e., they were perceived as interrupting the flow of the conversation more drastically). Nevertheless, it is worth highlighting that for both groups, the majority of TS were moderately natural (score = 2 on the 1–3 scale; see Figure 2 and Table 6). This suggests that autistic children in our sample did not exhibit entirely disruptive conversational behaviors, a finding which aligns with Ochs and Solomon's (2010) observations, who reported that autistic participants rarely produced radically incoherent utterances. Instead, they contributed utterances that were partially connected to the ongoing conversation but were not always considered fully relevant by their (neurotypical) interlocutors.

The present study provides a simple yet effective measure that can be applied in future research to systematically evaluate TS in conversation, addressing a gap in the literature (Ying Sng et al. 2018). Importantly, it does not only capture fine‐grained theoretical distinctions but also reflects people's perceptions, making it a valuable tool for identifying conversational atypicality. Moreover, our coding scheme can complement existing coding protocols (e.g., Abbot‐Smith et al. 2021), contributing to a more comprehensive framework for evaluating conversational dynamics.

### 4.3 Limitations and Future Research

A key limitation of this study concerns the sample. First, the relatively modest sample size limits the generalizability of our findings to the broader autistic population. To maximize sample size, we included both autistic males and females, but the low number of female autistic participants prevented separate analyses by sex. Second, the groups were not strictly matched on cognitive or language abilities. Since no delays were reported by schools or parents, it seems reasonable to assume that the neurotypical group would fall within the average range on standardized tests of receptive vocabulary and NVIQ, as was the case for the autistic group. Furthermore, the absence of significant group differences in MLU suggests a degree of comparability in language skills. Nevertheless, more rigorous matching would have strengthened the interpretability of group comparisons. Third, the limited data available on our participants hindered a deep examination of individual differences in topic management skills (Abbot‐Smith et al. 2023, 2024). Additionally, our study focused on experimenter‐child interactions. Future research could benefit from exploring how TS manifest across different interactional contexts (child–child conversations, familiar/unfamiliar interlocutors, mixed/non‐mixed dyads…). A further limitation concerns the rating task, which included a relatively small number of items per participant. While we opted for this number to keep the experiment within a manageable time frame and avoid participant fatigue, future research may consider increasing this number to further test the robustness of the present findings. Finally, given the mixed findings in the literature regarding how autistic adults judge unconventional conversational behaviors (Geelhand et al. 2021; Ying Sng et al. 2020), incorporating their perspectives could offer valuable insights into how conversational coherence is perceived or evaluated across neurotypes.

## 5 Conclusion

The present study aimed to investigate the topic management skills of Spanish‐speaking autistic and neurotypical children. Results showed no significant differences in their ability to maintain a conversation topic, with both groups producing a comparable number of contingent and minimal responses. However, autistic children produced significantly more TS, and these TS were perceived as more drastically interrupting the flow of the conversation than those produced by their neurotypical counterparts. On the basis of these findings, we conclude that while verbally fluent autistic children with average cognitive abilities are able to effectively engage in social interactions and elaborate on the interlocutor's previous topic, the frequency and nature of their TS may affect the extent to which their conversational behavior is perceived as coherent by their interlocutors, potentially leading to negative peer impressions. Importantly, the coding scheme employed in this study serves as a simple and systematic tool to code TS in conversation, which can be used in both research and clinical settings to better understand and support the conversational skills of autistic individuals.

## Funding

This research has been partially supported by project FUNLAT (PID2021‐122233OB‐I00), funded by MICIU/AEI, by “ERDF/EU,” the IT1537‐22 Research Group (Basque Government), the predoctoral grant from the Predoctoral Training Programme for Non‐Doctoral Research Staff of the Department of Science, Universities and Innovation of the Basque Government (PRE_2021_1_0008), and the FNRS‐F.R.S. WelChange Grant 40020204.

## Ethics Statement

All procedures performed in this study were approved by the Ethics Committee for research with human beings (CEISH) of the University of the Basque Country (UPV/EHU), code M10_2019_205. All parents/caregivers signed the consent form for the treatment of the data and the participation of their children in the study.

## Conflicts of Interest

The authors declare no conflicts of interest.

## Supporting information


**Data S1:** Supporting Information.

## Data Availability

The data that support the findings of this study are available in the Supporting Information of this article.
