# Supplementary material for: Conversational Topic Shifts and Topic Maintenance in Autistic and Neurotypical Children
Source: Autism Res. 2026 Feb 18;19(4):e70204. doi: 10.1002/aur.70204 (PMC13087834; doi:10.1002/aur.70204)
Supplement: Supplementary file 1 — Data S1: Supporting Information. [file AUR-19-0-s001.zip › Supplementary Materials/Supplementary Material 2_Rating task/Supplementary Material 2_Rating task.docx]

**Supplementary Material 2. Rating task**

To empirically support our coding scheme (see Supplementary Material 1) and ensure it captured relevant distinctions in how different topic shifts are perceived in conversation, we collected naturalness ratings from neurotypical adults (*N* = 96) via the online platform Prolific ([www.prolific.com](http://www.prolific.com)). The experiment lasted approximately 15 minutes and was programmed using PCIbex Farm (Zehr & Schwarz, 2018). Due to the novelty of the experiment, it was first pretested with 26 participants.

In this document, we present (1) the original instructions provided to participants in Spanish, along with their English translation; (2) the final set of items used in the main experiment; and (3) items initially included in the pretest that were later modified before the main experiment.

**1. Instructions**

**Spanish:**

| ¡Bienvenido/a a nuestro estudio!  Es muy sencillo y no te llevará mucho tiempo (15 minutos, aproximadamente). A continuación, leerás fragmentos de conversaciones espontáneas entre un adulto y un niño (de 6 a 12 años de edad). Estas conversaciones tuvieron lugar mientras el adulto y el niño jugaban a distintos juegos. Tu tarea es evaluar cómo de natural te parece la **intervención del niño** (marcada en **negrita**) en el contexto de la conversación. Algunas intervenciones serán más naturales que otras, por lo que es posible que algunas te resulten más inesperadas/extrañas. Utilizarás la escala del 1 al 7 para evaluar la intervención, donde 1 significa "nada natural" y 7 "completamente natural". Los valores intermedios (de 2 a 6) te permiten reflejar distintos grados de naturalidad. No hay respuestas correctas o incorrectas; solamente nos interesa tu intuición.  Antes de empezar, por favor, rellena el siguiente cuestionario y confirma tu consentimiento para participar.   \|  \| \| \| --- \| --- \| \| Edad: \|  \| \|  \| \| \| Género: \| Hombre  Mujer  No binario  Prefiero no decirlo  \| \|  \| \| \| Contacto habitual con niños/as: \| Sí  No  \| \|  \| \| \|  \| \| \| Confirmo que mi participación en este estudio es completamente voluntaria y que puedo abandonar el estudio en cualquier momento y por cualquier motivo, si así lo considero. Los datos obtenidos se asociarán a un número y nadie más tendrá acceso a ellos aparte del investigador en cuestión.  \| \| \| Acepto participar en este estudio.  \| \| \|  \| \| |
| --- | --- | --- | --- | --- | --- | --- | --- | --- | --- | --- | --- | --- | --- | --- | --- | --- | --- | --- | --- | --- | --- | --- |

Para continuar, tienes que aceptar participar en este estudio.

--

Haz click abajo para empezar con un pequeño entrenamiento (y no te preocupes, esta primera parte no forma parte del estudio).

Recuerda que tienes que leer la conversación e indicar cómo de natural te parece la **intervención** del niño (en **negrita**) en el contexto de la conversación.

Es importante que leas las conversaciones con atención, ya que puede haber preguntas adicionales acerca de estas.

*Training item*

En este caso, podríamos evaluar el enunciado del niño “Pues yo sí” como completamente natural, ya que no resulta extraño o inesperado en el contexto de la conversación.

Fácil, ¿verdad? Ahora vamos con el estudio. Presiona el botón para comenzar a leer.

**English** (translation):

Welcome to our study!

It’s very simple and won’t take much of your time (approximately 15 minutes). You will read excerpts from spontaneous conversations between an adult and a child (ages 6 to 12). These conversations took place while the adult and the child were playing different games. Your task is to evaluate how natural the **child’s utterance** (marked in **bold**) seems in the context of the conversation. Some utterances will be more natural than others, so some may seem more unexpected/strange. You will use a scale from 1 to 7 to evaluate the utterance, where 1 means “not natural at all” and 7 means “completely natural”. The intermediate values (2 to 6) allow you to reflect different degrees of naturalness. There are no right or wrong answers; we are only interested in your intuition.

Before starting, please fill out the following questionnaire and confirm your consent to participate.

|  | |
| --- | --- |
| Age: |  |
|  | |
| Gender: | Male  Female  Non-binary  Prefer not to say  |
|  | |
| Regular contact with children: | Yes  No  |
|  | |
|  | |
| I confirm that my participation in this study is completely voluntary, and I can leave the study at any time and for any reason, should I choose to do so. The data collected will be assigned a number, and no one other than the investigator will have access to it.  | |
| I agree to participate in this study.  To continue, you must agree to participate in this study.  --  Click below to begin with a short training (and don’t worry, this first part is not part of the study).  Remember that you need to read the conversation and indicate how natural the child’s **utterance** (in **bold**) seems in the context of the conversation.  It’s important that you read the conversations carefully, as there may be additional questions about them.  *Training item*  In this case, we could evaluate the child’s utterance as completely natural, since it doesn’t seem strange or unexpected in the context of the conversation.  Easy, right? Now let's move on to the study. Press the button to begin reading. | |

**2. Items of the main experiment (Spanish)**

**2.1. Training item**

*Adulto*: ¿Qué estación te gusta la que más?

*Niño*: Invierno o verano.

*Adulto*: ¿Invierno o verano?

*Niño*: Sí.

*Adulto*: A mí me gustan más la primavera y el otoño, porque es como que no hace mucho frío pero tampoco mucho calor. Porque yo el calor no lo soporto, la verdad.

*Niño*: **Pues yo sí.**

**2.2. Baseline items (no topic shifts)**

1.

*Adulto:* Vale, y cuando vas al monte de excursión, ¿qué cosas te llevas en la mochila?

*Niño:* Pues mira, bocadillos.

*Adulto:* Bocadillos. Importante, ¿no?

*Niño:* Sí. También agua, y un bifrutas.

*Adulto:* ¿Te gustan los bifrutas? ¿De qué te gustan? Porque hay distintos tipos, ¿no?

*Niño:* **El naranja. Ese es el mejor sabor.**

2.

*Adulto:* ¿Para qué necesitamos el botiquín en el monte?

*Niño:* Pues por si nos caemos, para curar la herida.

*Adulto:* Efectivamente, eso es. Imagínate que nos damos con una roca o así y empezamos a sangrar un poquito. Pues nos ponemos una tirita y ya está, ¿no?

*Niño:* Sí.

*Adulto:* ¿Tú te has caído alguna vez en el monte o en la calle?

*Niño:* **Sí, muchas veces. Creo que soy un poco torpe.**

3.

*Adulto:* ¿Tú has plantado nueces alguna vez?

*Niño:* Sí.

*Adulto:* ¿Y qué hay que hacer primero?

*Niño:* Un agujero.

*Adulto:* Un agujero, ¿no? ¿Y luego?

*Niño:* **Enterrarlas.**

4.

*Adulto:* ¿Qué te gusta hacer en tu tiempo libre?

*Niño:* Jugar al ajedrez.

*Adulto:* Ah, wow, me encanta el ajedrez. ¿Se te da bien?

*Niño:* Sí, se me da bien.

*Adulto:* ¿Llevas mucho tiempo jugando?

*Niño:* **Sí, tres años ya.**

**2.3. Critical items**

**Condition 1 – associated explicit none**

List A

1.

*Adulto:* A mí me encanta la leche. Yo tomo un montón de leche.

*Niño:* Yo tomo yogures.

*Adulto:* Yogures también, y queso. Me encanta el queso.

*Niño:* Pues a mí no.

*Adulto:* ¿No? ¿Y qué te gusta?

*Niño:* **A mí me gusta el chocolate, las patatas… Mira, pues yo hoy me voy a comer patatas a la patatería de la Plaza España.**

2.

*Adulto:* Ah, ¿vives en San Miguel?

*Niño:* Sí.

*Adulto:* Ah, ya sabía yo.

*Niño:* ¿Y tú?

*Adulto:* Yo aquí, cerca del ayuntamiento.

*Niño:* **Ya. ¿Sabes cómo llama mi madre a los ayuntamientos?**

List B

1.

*Adulto:* ¿En el zoo viste ardillas?

*Niño:* No, pero si las hubiera visto les hubiera dado una nuez.

*Adulto:* Les darías una nuez, claro. Las bellotas también les gustan.

*Niño:* Sí, pero sobre todo las nueces.

*Adulto:* Sobre todo, sí.

*Niño:* **Aparte, son muy difíciles de abrir.**

2.

*Adulto:* Mira esto.

*Niño:* Un botiquín de emergencias.

*Adulto:* Muy bien.

*Niño:* Por si me pica una serpiente.

*Adulto:* Vale, a ver, esperemos no encontrarnos ninguna serpiente en el monte pero bueno.

*Niño:* **Esperemos. Oye, ¿sabes qué podemos encontrarnos? Perros.**

**Condition 2 – associated explicit question**

List A

1.

*Adulto:* Puedes terminar alguno de estos dibujos o dibujar algo nuevo por detrás.

*Niño:* ¿Tienes un dibujo de un león?

*Adulto:* Mira, sí. ¿Quieres terminar este?

*Niño:* Sí.

*Adulto:* ¿Sí? ¿Te apetece? Venga, yo voy a coger este de la niña y el perro. ¿Y qué quieres, rotus o pinturas?

*Niño:* **Espera, pero antes de empezar, he visto un dibujo que me ha gustado.**

2.

*Adulto: ¿*Tú cuando vas al monte qué cosas te llevas en tu mochila?

*Niño:* En realidad no sé ni lo que llevo…

*Adulto:* A ver, ¿tú qué te llevarías al monte?

*Niño:* Una cantimplora.

*Adulto:* Una cantimplora, súper importante. Para beber agua, ¿no? ¿Y qué más?

*Niño:* **Y hablando de sed, me está entrando algo de sed.**

List B

1.

*Adulto: ¿*Cuál es tu animal favorito de estos?

*Niño:* Los caballos y las vacas no me van mucho.

*Adulto:* ¿Cuál es tu animal favorito entonces, a ver? ¿Tienes alguno favorito?

*Niño:* Mmm, no.

*Adulto:* ¿No puedes elegir?

*Niño:* **Te digo una cosa. Hay vacas que ¿sabes qué tienen a veces?**

2.

*Adulto:* Me encantaría ir a Madagascar, pero nunca he ido.

*Niño:* Pues si vas, que te diviertas con tus veintiseis horas en avión.

*Adulto:* ¿Tú has ido alguna vez? ¿Cuál es el viaje más largo que has hecho?

*Niño:* El viaje más largo que he hecho es a las Islas Canarias, a Gran Canaria.

*Adulto:* ¿A Gran Canaria? Yo estuve en Tenerife el año pasado. Es muy bonito Canarias, ¿no? ¿Te gustó?

*Niño:* **Pues hemos pensado esta navidad ir a Egipto.**

**Condition 3 – associated explicit statement**

List A

1.

*Adulto:* Oye, y si este árbol ha dado estas nueces, ¿qué tipo de árbol crees que es?

*Niño:* No lo sé.

*Adulto:* Esto es un nogal. Los nogales dan nueces. ¿Te gustan las nueces?

*Niño:* Sí.

*Adulto:* ¿Sí? Jo, mi abuela tiene una huerta, y bueno, aparte de verduras y eso también me suele dar un montón de nueces.

*Niño:* **¿Pues sabes? Creo que he encontrado una mala.**

2.

*Adulto:* ¿Dibujas mucho en casa?

*Niño:* Mmm, un poquito.

*Adulto:* ¿Sabes que a mí se me da fatal dibujar? No sé hacer ni una casa.

*Niño:* Te has salido.

*Adulto:* ¿Has visto? Si es que… Bueno, he de decir que esto lo ha hecho otro niño. Pero sí, no se me da muy bien.

*Niño:* **Mira, el truco para que se vea más es este.**

List B

1.

*Adulto:* ¿Fuiste a montar a caballo?

*Niño:* Sí.

*Adulto:* ¿Y cuántas veces has montado? ¿Te gusta montar a caballo?

*Niño:* Sí, una vez. Mi madre me enseñó cómo hacerlo.

*Adulto:* Yo una vez me monté en uno. El caballo se puso súper nervioso y empezó a correr conmigo encima. Ya no me vuelvo a montar.

*Niño:* **Pues también existen los caballos blancos.**

2.

*Adulto:* ¿Qué es esto?

*Niño:* ¡Nueces!

*Adulto:* ¿Te gustan las nueces?

*Niño:* A mí bueno, pero a papá y a mamá les gustan para la ensalada.

*Adulto:* ¿Para la ensalada? Bua, están riquísimas. Yo también le echo nueces a la ensalada, y a veces cacahuetes también.

*Niño:* **Mira, ¡he cazado una nuez!**

**Condition 4 – associated implicit none**

List A

1.

*Adulto:* ¿Tenías ganas ya de empezar el cole o preferías quedarte de vacaciones un poco más?

*Niño:* Tenía ganas para estar con mis amigos.

*Adulto:* Claro. ¿No les has visto en verano?

*Niño:* No. Bueno, cuando acabó el verano vi a mi amigo Aitor.

*Adulto:* Ah, qué guay.

*Niño:* **¿Conoces a alguien que se llame Aitor?**

2.

*Adulto:* ¿Para qué necesitamos un botiquín en el monte?

*Niño:* Porque igual te puedes hacer daño. Yo nunca me he hecho daño.

*Adulto:* ¿No? Pues qué suerte, eh.

*Niño:* Pues sí.

*Adulto:* Vale, pues vamos a guardar todo en la mochila. A ver si consigo cerrar el botiquín. ¿Puedes guardar las galletas mientras?

*Niño:* **Sí, vale, a ver. Yo estas galletas las comí hace no mucho para desayunar.**

List B

1.

*Adulto:* ¿Nunca te has ido de excursión al monte?

*Niño:* Sí, alguna vez sí.

*Adulto:* A mí me gusta mucho ir al monte.

*Niño:* ¿Sí?

*Adulto:* Sí, yo voy mucho al monte. ¿Sabes dónde está el Gorbea?

*Niño:* **Sí. Yo siempre voy al monte en coche.**

2.

*Adulto:* Oye, ¿y tus amigas han venido a acompañarte?

*Niño:* Sí, y porque mi padre les ha invitado a comer unas patatas cuando terminemos.

*Adulto:* Ah, ¿y eso por qué? ¿Celebráis algo?

*Niño:* No, pero él sabe que nos encantan.

*Adulto:* Qué majo, ¿no? ¿Cómo se llama tu padre?

*Niño:* **Carlos. ¿Tú te llamabas Sara?**

**Condition 5 – associated implicit question**

List A

1.

*Adulto:* Yo creo que les pasa algo a estos animales. Están un poco tristes.

*Niño:* El burrito tiene la cabeza agachada. Está llorando.

*Adulto:* ¿Está llorando? ¿Por qué?

*Niño:* No sé.

*Adulto:* ¿Tú crees que le dolerá alguna pata?

*Niño:* **El caballo ya la tiene curada.**

2.

*Adulto:* A ver, vamos a llegar a un sitio…

*Niño:* ¡Hay animales!

*Adulto:* Wow, ¡cuántos animales!

*Niño:* ¡Ya!

*Adulto:* A ti te gustaban mucho los animales, ¿no?

*Niño:* **Esta vaca tiene una herida.**

List B

1.

*Adulto:* ¿Tú cuando vas al monte qué cosas te llevas en la mochila?

*Niño:* Pues no llevo nada.

*Adulto:* ¿Nada?

*Niño:* No llevo mochila.

*Adulto:* ¿No llevas mochila al monte?

*Niño:* **Mi mochila es de Decathlon.**

2.

*Adulto:* ¿Sabes lo que tengo yo en casa?

*Niño:* ¿El qué?

*Adulto:* Tengo un gato.

*Niño:* ¿Cómo es?

*Adulto:* Es gris. Tiene un montón de pelo, parece una bola de pelo. ¿Sabes cuáles son los gatos persa?

*Niño:* **Tengo una amiga que se llama Toñi que tiene un gato. Es solo naranja y tiene pelo también.**

**Condition 6 – associated implicit statement**

List A

1.

*Adulto:* Venga, ¿dibujas ahí detrás algo?

*Niño:* Sí.

*Adulto:* ¿Qué prefieres, rotus o pinturas?

*Niño:* Rotus.

*Adulto:* ¿Rotus? Pues coge. Yo voy a terminar este dibujo.

*Niño:* **Tengo más rotus que pinturas en casa.**

2.

*Adulto:* Vale, y una última cosa…

*Niño:* La brújula.

*Adulto:* Muy bien. ¿Para qué sirve la brújula?

*Niño:* Para decirnos el sur y el norte.

*Adulto:* Eso es, y también el este y el oeste. Vale, para no perdernos es muy importante.

*Niño:* **Mi amigo Joel tiene este reloj, pero en brújula. En color dorado, con distintos dibujos.**

List B

1.

*Adulto:* ¿Te gusta dibujar?

*Niño:* Mmm, bueno…

*Adulto:* ¿Más o menos?

*Niño:* Sí. ¿Qué tengo que dibujar?

*Adulto:* Tienes dos opciones. Puedes terminar un dibujo que ya está empezado o hacer uno nuevo. Como tú quieras.

*Niño:* **La gente dibuja muy bien.**

2.

*Adulto:* ¿Tú alguna vez has plantado algo?

*Niño:* En el cole plantamos guisantes y más cosas.

*Adulto:* A mí no me gustan mucho los guisantes.

*Niño:* Ni a mí.

*Adulto:* Mi abuela tiene una huerta, y planta pimientos y así. Luego le salen más, y así no tiene que comprar.

*Niño:* **Nuestra profesora tiene un huerto, y le picó un mosquito en el brazo, o una abeja.**

**Condition 7 – topic reintroduction explicit none**

List A

1.

*Adulto:* ¿Cómo se llama tu petirrojo?

*Niño:* Se llama, eh… necesito pensar.

*Adulto:* ¿Se te ha olvidado con tantos nombres? No pasa nada, es normal. ¿Tienes también hermanos o así? ¿O eres hijo único?

*Niño:* Hijo único. Tengo primas y primos.

*Adulto:* Vale, así que tu familia es bastante grande.

*Niño:* **Eh, sí. Eh ya me acuerdo de cómo se llama. Se llama Ruisi.**

2.

*Adulto:* No creo que erupcione ese volcán, no creo.

*Niño:* ¡Sí va a erupcionar!

*Adulto:* Mira, vamos a ver esta granja ahora, ¿vale? ¿Qué animales hay en esta granja, a ver?

*Niño:* Un caballo, un burro, una vaca y una cebra.

*Adulto:* A mí me encantan las cebras. ¿Has visto alguna vez una cebra?

*Niño:* **Sí. ¿Sabes dónde está el volcán? Aquí al lado.**

List B

1.

*Adulto:* ¿Cuál es tu animal favorito?

*Niño:* Los gatos y los monos. Los monos los vi en el zoo.

*Adulto:* Ah, qué guay. ¿Y te gustan los perros?

*Niño:* Mmm, sí, me gustan.

*Adulto:* Ah, vale.

*Niño:* **¿Pues sabes? ¿Alguna vez has ido al zoo?**

2.

*Adulto:* Venga pues vamos a meter todas las cosas en la mochila antes de que se nos haga de noche. Venga, vámonos de excursión. Oye, ¿cuál es tu animal favorito?

*Niño:* Los perritos.

*Adulto:* ¿Tienes alguno en casa?

*Niño:* No, porque mi madre es alérgica.

*Adulto:* No quiere, ¿no? Claro.

*Niño:* ***¡*Venga, venga, ya es de noche!**

**Condition 8 – topic reintroduction explicit question**

List A

1.

*Adulto:* Ah, no conozco esa zona de Galicia. Yo he ido una vez a Santiago. ¿Has estado?

*Niño:* Mmm, no sé.

*Adulto:* También está en Galicia. ¿Y te gusta ir allí a pasar el verano con la abuela?

*Niño:* Sí. Me gusta ir a la piscina.

*Adulto:* ¿Tiene piscina en su casa?

*Niño:* **Y también Pontevedra.**

2.

*Adulto:* A ver, pintar se me da bien, pero dibujar como tú estás haciendo se me da fatal. ¿Has dibujado un árbol?

*Niño:* Sí.

*Adulto:* No te chupes. ¿Quieres papel?

*Niño:* Me pica. Me ha picado un mosquito.

*Adulto:* Ah, ¿sí? Ya con el calor empiezan a salir mosquitos… Oye, por cierto, ¿tú haces algún deporte?

*Niño:* **Espera, que acabo el árbol.**

List B

1.

*Adulto:* El rotulador gris lo tengo yo. ¿Te lo dejo?

*Niño:* Sí, sí, sí.

*Adulto:* Bueno, me decías que habías estado con Julia también. ¿Te lo pasaste bien con ella?

*Niño:* Sí, muy bien.

*Adulto:* Oye, y estos días que no tienes cole, ¿qué estás haciendo en casa?

*Niño:* **Espera, un poco más de gris.**

2.

*Adulto:* ¿Para qué necesitamos la brújula en el monte?

*Niño:* Para no perdernos. Yo la tengo en mi bastón.

*Adulto:* ¿Tu bastón tiene brújula? Mira, otra cosa que se nos había olvidado, bastones para el monte.

*Niño:* Sí. Y también suelo llevar zapatillas por si acaso. Y paraguas también.

*Adulto:* Ah, el paraguas lo tengo aquí. Lo que no he traído ha sido bastones, pero bueno. Oye, qué guay que tus bastones tengan una brújula, ¿no?

*Niño:* **Y visera o gafas de sol.**

**Condition 9 – topic reintroduction explicit statement**

List A

1.

*Adulto:* ¿A dónde sueles ir con tus amigos?

*Niño:* Eh bueno, es que claro como no conoces Vitoria… Aunque igual mi calle la conoces. ¿Sabes cuál es la calle peatonal? Yo vivo ahí, en un edificio con once alturas.

*Adulto:* Sí, sé cuál es. Ah, yo vivo en el séptimo piso.

*Niño:* Yo en el cuarto.

*Adulto:* En el cuarto, mhm. Venga, vamos a dibujar un dibujo que tenga que ver con la excursión de hoy.

*Niño:* **A ver, ah, también tenía que decirte lo de mis amigos.**

2.

*Adulto:* Mira la mochila que he preparado yo. A ver qué te parece. ¿Eso qué es?

*Niño:* Un botiquín.

*Adulto:* Por si nos hacemos alguna herida, ¿no? O por si hay algún accidente. ¿Tú alguna vez has tenido un accidente de excursión?

*Niño:* No, no recuerdo.

*Adulto:* Yo una vez sí. Me acuerdo que fui de excursión a una granja, íbamos en bici y me caí de la bici por una ladera en la montaña.

*Niño:* **Y esto es una linterna, ¿no?**

List B

1.

*Adulto:* Vas a hacer genial el examen. ¿Es fácil?

*Niño:* Pues sí. Estamos ensayando los prefijos ‘des’ e ‘in’ y los determinantes numerales.

*Adulto:* Qué guay, a mí me encanta. Yo estudié lingüística. ¿Sabes lo que es?

*Niño:* Sí, lo sé.

*Adulto:* Pues yo estudié eso. Yo soy lingüista.

*Niño:* **Ah, y también el prefijo ‘ir’.**

2.

*Adulto:* ¿A qué monte has ido?

*Niño:* Bueno, está cerca de Landa.

*Adulto:* Oye, ¿y tú qué cosas te llevas en la mochila cuando vas al monte?

*Niño:* Almuerzo.

*Adulto:* Ah, un bocata de tortilla has dicho antes.

*Niño:* **Y he ido a Barria.**

**Condition 10 – topic reintroduction implicit none**

List A

1.

*Adulto:* ¿Qué le ha pasado a este árbol?

*Niño:* Se le han caído todas las hojas y nueces por el otoño.

*Adulto:* Eso es, en otoño se caen las hojas. Tu cumple es en verano, ¿no?

*Niño:* Sí.

*Adulto:* Mi cumple es en otoño, cerca de Halloween.

*Niño:* **Ya. ¿Cómo pongo las nueces?**

2.

*Adulto:* Venga, vamos. Mira, estamos en el monte.

*Niño:* Hay hojas caídas.

*Adulto:* Hala, hay un montón de hojas en el suelo.

*Niño:* Y eso.

*Adulto:* ¿Eso qué es?

*Niño:* **Castañas. Vamos a colocar las hojas en el árbol.**

List B

1.

*Adulto:* Vale, ¿qué color quieres? Yo voy a coger el marrón para acabar con el perro. ¿Tú el verde para el árbol?

*Niño:* Sí.

*Adulto:* ¿Me has dicho que hacías papiroflexia? ¿Eso qué es?

*Niño:* Bueno, doblar…

*Adulto:* Ah, vale, lo de hacer figuras con…

*Niño:* **Sí. Mejor cojo rojo.**

2.

*Adulto:* ¿Qué le ha pasado a este árbol?

*Niño:* Se le han caído todas las hojas y nueces por el otoño.

*Adulto:* Eso es, en otoño se caen las hojas. ¿Cuál es tu estación favorita?

*Niño:* El verano, por la playa. Voy con mi familia y está arriba de una montaña.

*Adulto:* ¿El qué? ¿La playa?

*Niño:* **Sí. ¿Coloco las hojas?**

**Condition 11 – topic reintroduction implicit question**

List A

1.

*Adulto:* Oye, ¿tú haces judo?

*Niño:* Sí.

*Adulto:* Ah, haces judo. ¿Y qué tal? ¿Te gusta?

*Niño:* No, no me gusta.

*Adulto:* ¿Qué te gusta hacer?

*Niño:* **Solo hay peleas.**

2.

*Adulto:* Vivo en Basauri, cerca de Bilbao. ¿Has estado en Bilbao alguna vez?

*Niño:* Sí, algunas veces. He ido a la playa, al cohete…

*Adulto:* ¿Al cohete?

*Niño:* Sí.

*Adulto:* ¿Qué cohete?

*Niño:* **¿Por qué se llama Basauri?**

List B

1.

*Adulto:* ¿Has plantado Lacasitos?

*Niño:* Sí, un Lacasito.

*Adulto:* ¿En serio? Bua, sería genial un árbol de Lacasitos, ¿verdad?

*Niño:* Sí.

*Adulto:* Bueno, ya hemos plantado las nueces, ¿verdad?

*Niño:* **¿A que si plantamos un Lacasito saldrá otro?**

2.

*Adulto:* ¿Qué le ha pasado a este árbol?

*Niño:* Se le han caído las hojas.

*Adulto:* ¿Por qué se caen? Porque es…

*Niño:* Otoño.

*Adulto:* Otoño, muy bien, porque es otoño. ¿Y cuántas hojas hay?

*Niño:* **Es el último día de otoño.**

**Condition 12 – topic reintroduction implicit statement**

List A

1.

*Adulto:* ¿Qué saldrá si plantamos una nuez?

*Niño:* Un árbol de nueces.

*Adulto:* Eso es. Venga, ayúdame a plantarlas. ¡Súper bien!

*Niño:* Ya está.

*Adulto:* Ahora lo que vamos a hacer es fijarnos en estos animales.

*Niño:* **Las nueces están malas.**

2.

*Adulto:* Qué original el dibujo.

*Niño:* Pues dame otro folio.

*Adulto:* ¿Otro folio? Mmm, se me ocurre otra cosa mejor. Vamos a ver un vídeo en mi móvil, ¿vale? Yo voy a guardar el dibujo para ponerlo en mi casa, que me ha gustado mucho. ¿Me dejas?

*Niño:* Sí.

*Adulto:* Tengo un vídeo que te va a gustar.

*Niño:* **¿Tienes otro folio para dibujar?**

List B

1.

*Adulto:* Muy bien, se han caído las hojas porque es otoño. Y también vemos nueces, ¿no? ¿Qué podemos hacer con estas nueces?

*Niño:* Recogerlas.

*Adulto:* Oye, ¿tú sabes a qué animal le gustan mucho las nueces?

*Niño:* No sé.

*Adulto:* A las ardillas. A las ardillas les encanta comer nueces.

*Niño:* **En otoño hace frío.**

2.

*Adulto:* Mira, yo tengo dibujos aquí. Puedes pintar el del león, la niña con el perro, los tiburones…

*Niño:* Pues los tiburones.

*Adulto:* Vale.

*Niño:* Me encantan los leones también.

*Adulto:* A mí también. ¿Sabes que hace poco vi un león? Lo vi en el zoo.

*Niño:* **¿Los tiburones de qué color son? ¿Grises?**

**Condition 13 – non-associated explicit none**

List A

1.

*Adulto:* Mira, puedes elegir entre pinturas o rotus.

*Niño:* Yo prefiero lápiz.

*Adulto:* Vale, ¿entonces este o lápiz de cuál?

*Niño:* Lápiz de los normales.

*Adulto:* Toma. A ver, ¿qué estás dibujando? Ah, somos nosotros.

*Niño:* **Sí. ¿Tú sabes que en las selvas de África hay un montón de cebras?**

2.

*Adulto:* Hay animales que también vuelan, eh.

*Niño:* Ah, mira, el pájaro.

*Adulto:* Bueno, ¿cuál es tu animal favorito?

*Niño:* El búho.

*Adulto:* Oh, el búho con los ojos gigantes.

*Niño:* **Sí, con los ojos gigantes. Mira, con las hojas se hace esto.**

List B

1.

*Adulto: ¿*Estuviste en el monte Gorbea?

*Niño:* Sí.

*Adulto:* Qué guay, ¿con quién?

*Niño:* Con muchos profesores.

*Adulto:* Ah, ¿con el cole? Pensaba que habías ido con tu familia.

*Niño:* **Sí. Eh, mira, yo tengo un juego que se llama ‘cámara espía’**.

2.

*Adulto:* Ah, ¿tu tío tiene un gatito también?

*Niño:* Sí.

*Adulto:* ¿Sabes cómo es mi gata?

*Niño:* ¿Cómo?

*Adulto:* Es gris y tiene un montón de pelo. Parece una bola de pelo.

*Niño:* **Sí, lo parece. Mira esta nuez.**

**Condition 14 – non-associated explicit question**

List A

1.

*Adulto:* Mira, lo que tengo. ¿Qué es esto?

*Niño:* La brújula. Te enseña el norte, el sur, el este y el oeste.

*Adulto:* ¿Y tú sabes usarla?

*Niño:* Pues no, porque no sé en qué dirección está mi casa.

*Adulto:* Claro. Vale, venga, vámonos al monte. ¿Quieres llevar tú la mochila o la llevo yo?

*Niño:* **Ay, una cosa. ¿Sabes una de las cosas que más miedo me dan? Las alturas.**

2.

*Adulto:* Bueno, vamos con tus padres.

*Niño:* Vale, y si puedo voy a comerme un Kinder.

*Adulto:* Yo no tengo Kinder, eh.

*Niño:* Ya, los Kinders están en la cafetería.

*Adulto:* Ah, los has fichado ya. Bueno, ¿te lo has pasado bien?

*Niño:* **Oye, perdona porque tengo que quitar las hojas.**

List B

1.

*Adulto:* ¿Tú te has roto alguna vez algo?

*Niño:* No, pero mi hermano sí. Se ha roto el brazo dos veces.

*Adulto:* ¿Qué dices? Pobrecillo. Bueno, volvemos a la actividad. ¿Ya está recuperada la cebra?

*Niño:* Sí.

*Adulto:* Muy bien. Venga, vamos a dejar aquí a nuestros amigos los animales. Nos despedimos ya de ellos, ¿vale?

*Niño:* **Mira, ¿sabes qué? Mira, hay un videojuego que se llama Minecraft. ¿Sabes cuál es?**

2.

*Adulto:* ¿Qué le pasa a este árbol?

*Niño:* Le faltan estas hojas.

*Adulto:* ¿Por qué crees que le faltan hojas a este árbol?

*Niño:* ¿Porque se le han caído?

*Adulto:* Muy bien, se le han caído, ¿no? ¿Y cuándo se caen las hojas de los árboles?

*Niño:* **A ver, ¡nos hemos perdido!**

**Condition 15 – non-associated explicit statement**

List A

1.

*Adulto:* Pues ahora nos quedan dos cositas.

*Niño:* ¿Cuáles?

*Adulto:* Hacer un dibujo…

*Niño:* ¿Puede ser de animales?

*Adulto:* Tiene que ser algo relacionado con lo que hemos hecho hoy.

*Niño:* **Ay, se ha salido una nuez.**

2.

*Adulto:* Oye, ¿cuál es tu estación favorita del año? ¿Cuál te gusta más?

*Niño:* El verano.

*Adulto:* ¿El verano? ¿Por qué?

*Niño:* Porque hace mucho sol.

*Adulto:* Jo, pues a mí me agobia un poco tanto calor.

*Niño:* **Mira, mira. Hola, oveja.**

List B

1.

*Adulto:* ¿Qué te gusta hacer a ti en tu tiempo libre?

*Niño:* Jugar a la play.

*Adulto:* ¿Qué juegos tienes en la play?

*Niño:* El Among Us, el Minecraft…

*Adulto:* Hala, yo nunca he jugado al Among Us.

*Niño:* **Uy, una diana.**

2.

*Adulto:* Con las bolas es más difícil acertar. ¿Quieres probar con un dardo?

*Niño:* Sí.

*Adulto:* Acércate un poco más.

*Niño:* Pues mira, al diez justo.

*Adulto:* ¡Al diez! Te he ganado entonces.

*Niño:* **Mira, una cebra.**

**Condition 16 – non-associated implicit none**

List A

1.

*Adulto:* Pues a mí me gusta más la primavera que el verano.

*Niño:* A mí no.

*Adulto:* ¿Qué sueles hacer en verano? ¿Te sueles ir de vacaciones?

*Niño:* Mhm.

*Adulto:* ¿Sí?

*Niño:* **Sí. Voy a abrir la nevera.**

2.

*Adulto:* ¿Qué animales tienes?

*Niño:* Tengo un gato y un perro.

*Adulto:* Ah, yo tengo un gato también.

*Niño:* ¿Y un perro?

*Adulto:* No, un perro no, un gato. Tengo un gato persa. ¿Sabes esa raza cuál es?

*Niño:* **Sí. Vamos a jugar con eso ya.**

List B

1.

*Adulto:* ¿No sabes cómo son las nueces por dentro?

*Niño:* No.

*Adulto:* Jo, pues no te sé explicar. Como mi abuela tiene, un día te traigo una de verdad y te la enseño.

*Niño:* Sí.

*Adulto:* ¿Vale?

*Niño:* **Sí. Te conozco desde hace mucho tiempo.**

2.

*Adulto:* A ver dónde puedes pintar algo.

*Niño:* ¿Esos dibujos quién los hizo?

*Adulto:* Más niños.

*Niño:* Y se lo pasaron bien, ¿no?

*Adulto:* Claro. ¿Tú te lo estás pasando bien?

*Niño:* **Sí. Pájaro, ¿qué haces en mi mano?**

**Condition 17 – non-associated implicit question**

List A

1.

*Adulto:* ¿Tenéis una casa muy grande?

*Niño:* Eh, sí.

*Adulto:* Oye, ¿y cuando fuiste de acampada qué cosas te llevaste?

*Niño:* Pues llevé un helado.

*Adulto:* ¿Un helado? Me encantan los helados. ¿De qué te gusta a ti el helado?

*Niño:* **Luego durante el sueño tenía calor, pero cuando me desperté tuve frío.**

2.

*Adulto:* Venga, este va a ser nuestro monte de hoy. ¿Te parece?

*Niño:* Vale.

*Adulto:* ¿Quién lleva la mochila? ¿Tú o yo?

*Niño:* Yo.

*Adulto:* ¿Conoces algún monte famoso o así? ¿Me puedes decir algún monte al que hayas ido?

*Niño:* **¿Qué es este dibujo?**

List B

1.

*Adulto:* ¿A ti qué te gusta más, ir al monte o leer libros?

*Niño:* Leer libros.

*Adulto:* Ah, muy bien. Oye, por cierto, ¿sabes de dónde es tu gorra?

*Niño:* ¿De Cataluña?

*Adulto:* No. Es de Sopelana, ¿no? ¿Sabes dónde está Sopelana?

*Niño:* **Mi profesora siempre me deja el ordenador cuando he trabajado.**

2.

*Adulto:* ¿Y al perrito de tus abuelos también le ves mucho?

*Niño:* Sí.

*Adulto:* ¿Se lleva bien con tu gato o no?

*Niño:* Pero mi gato no va a la casa de los abuelos.

*Adulto:* Ah, nunca ha ido. ¿Nunca se han conocido entonces?

*Niño:* **¿Qué celo tiene más cinta?**

**Condition 18 – non-associated implicit statement**

List A

1.

*Adulto:* Bueno, ¿y qué tal con lo de la vuelta al cole? ¿Tienes ganas o no?

*Niño:* No.

*Adulto:* No, ¿verdad? Da un poco de pereza.

*Niño:* Sí.

*Adulto:* Otra vez a madrugar, otra vez deberes…

*Niño:* **Ahora está trabajando mamá.**

2.

*Adulto:* ¿Tú te has ido de excursión al monte alguna vez?

*Niño:* No.

*Adulto:* Pues vamos a ir al monte de excursión hoy. ¿Qué tengo yo aquí?

*Niño:* Una mochila.

*Adulto:* Vale, luego vamos a ver lo que tengo en la mochila. La primera prueba es que tienes que buscar cuatro animales que están escondidos por la sala.

*Niño:* **¿Esta nuez es de verdad?**

List B

1.

*Adulto:* Vale, es otoño hemos dicho, ¿verdad?

*Niño:* Sí.

*Adulto:* Tu cumple es en verano, ¿no?

*Niño:* Sí.

*Adulto:* Mi cumple es en otoño.

*Niño:* **¿Cómo pongo esto?**

2.

*Adulto:* Efectivamente, ¿cómo sabías que la brújula estaba en inglés?

*Niño:* Porque aquí pone una uve doble.

*Adulto:* ¿Sabes cómo se dice en inglés ‘oeste’?

*Niño:* ¿Cómo?

*Adulto:* West.

*Niño:* **Hay que abrir las nueces.**

**2.4. Attention checks**

Each list included two attention checks to ensure participants were paying attention to the task. The attention checks consisted of multiple-choice questions about the content of the preceding conversation. For example:

¿Sobre qué era la conversación anterior? Selecciona la respuesta correcta.

*A: Sobre montes.*

*B: Sobre dibujos.*

*C: Sobre nueces.*

**3. Items of the main experiment (translated into English)**

**3.1. Training item**

*Adult*: Which season do you like the most?

*Child*: Winter or summer.

*Adult*: Winter or summer?

*Child*: Yes.

*Adult*: I like spring and fall best, because it’s not too cold but not too hot either. Because I can’t stand the heat, to tell you the truth.

*Child*: **Well, I can.**

**3.2. Baseline items (no topic shifts)**

1.

*Adult:* Okay, and when you go hiking, what do you carry in your backpack?

*Child:* Well, a sandwich.

*Adult:* A sandwich. Important, isn’t it?

*Child:* Yes. Also water and juice.

*Adult:* Do you like juice? What flavors do you like? Because there are different kinds, right?

*Child:* **Orange. That’s the best flavor.**

2.

*Adult:* What do we need a first-aid kit for in the mountain?

*Child:* Well, in case we fall, to heal the wound.

*Adult:* That’s right. Imagine we hit a rock or something and we start to bleed a little bit. We put a band-aid on and that’s it, right?

*Child:* Yes.

*Adult:* Have you ever fallen in the mountain or in the street?

*Child:* **Yes, many times. I think I’m a bit clumsy.**

3.

*Adult:* Have you ever planted walnuts?

*Child:* Yes.

*Adult:* And what do we have to do first?

*Child:* A hole.

*Adult:* A hole, right? And then?

*Child:* **Cover them.**

4.

*Adult:* What do you like doing in your free time?

*Child:* Playing chess.

*Adult:* Ah, wow, I love chess. Are you good at it?

*Child:* Yes, I’m good at it.

*Adult:* Have you been playing for a long time?

*Child:* **Yes, three years now.**

**3.3. Critical items**

**Condition 1 – associated explicit none**

List A

1.

*Adult:* I love milk. I drink a lot of milk.

*Child*: I eat yogurt.

*Adult:* Yogurts too, and cheese. I love cheese.

*Child:* I don’t.

*Adult:* No? And what do you like?

*Child:* **I like chocolate, French fries… Look, today I’m going to eat French fries in Plaza España.**

2.

*Adult*: Oh, do you live in San Miguel?

*Child*: Yes.

*Adult*: I knew it.

*Child*: And you?

*Adult*: I live here, near the town hall.

*Child*: Ah. **Do you know how my mom refers to town halls?**

List B

1.

*Adult:* Did you see squirrels at the zoo?

*Child:* No, but if I had seen them I would have given them a walnut.

*Adult:* You would give them a walnut, of course. They like acorns, too.

*Child:* Yes, but they like walnuts more.

*Adult:* That’s true, yes.

*Child:* **Besides, they are very hard to open.**

2.

*Adult:* Look at this.

*Child:* An emergency kit.

*Adult:* Very good.

*Child:* In case I get bitten by a snake.

*Adult:* Okay, let’s hope we don’t find any snakes in the mountain, but okay.

*Child:* **Let’s hope so. Hey, do you know what we might find? Dogs.**

**Condition 2 – associated explicit question**

List A

1.

*Adult:* You can finish one of these drawings or draw something new on the back.

*Child:* Do you have a drawing of a lion?

*Adult:* Look, yes. Do you want to finish this one?

*Child:* Yes.

*Adult:* Yes? Do you feel like it? Come on, I’ll take this one of the girl and the dog. And what do you want, markers or crayons?

*Child:* **Wait, but before I start, I just saw a drawing I liked.**

2.

Adult: When you go hiking, what do you carry in your backpack?

Child: Actually, I don’t even know what I take…

Adult: Okay, what would you take with you when you go hiking?

Child: A canteen.

Adult: A canteen to drink water, right? What else?

Child: **And** **speaking of thirst, I’m starting to feel a bit thirsty.**

List B

1.

*Adult:* What is your favorite animal?

*Child:* I don’t like horses and cows much.

*Adult:* What’s your favorite animal, then? Let’s see, do you have a favorite?

*Child:* Mmm, no.

*Adult:* You can’t choose?

*Child:* **I’ll tell you what. There are cows that do you know what they have sometimes?**

2.

*Adult:* I’d love to go to Madagascar, but I’ve never been.

*Child:* Well, if you go, enjoy the twenty-six hours on the plane.

*Adult:* Have you ever been there? What is the longest trip you’ve ever been on?

*Child:* The longest trip I’ve ever been on was to the Canary Islands, to Gran Canaria.

*Adult:* To Gran Canaria? I was in Tenerife last year. It’s very nice, isn’t it? Did you like it?

*Child:* **Well, this Christmas we are thinking of going to Egypt.**

**Condition 3 – associated explicit statement**

List A

1.

*Adult*: Hey, what kind of tree do you think it is, since it has walnuts?

*Child*: I don’t know.

*Adult*: This is a walnut tree. Walnut trees bear walnuts. Do you like walnuts?

*Child*: Yes.

*Adult*: Yes? My grandma has a vegetable garden, and she gives me loads of walnuts every time I visit her.

*Child*: **You know what? I think I found one that’s spoiled.**

2.

*Adult:* Do you draw a lot at home?

*Child:* Mmm, a little bit.

*Adult:* Did you know that I’m terrible at drawing? I can’t even draw a house.

*Child:* You’ve painted outside the line.

*Adult:* You see? Well, I have to say that this was done by naother child. But yes, I’m not very good at it.

*Child:* **Look, here’s the trick to make it stand out more.**

List B

1.

*Adult:* Did you go for a horse ride?

*Child:* Yes.

*Adult:* How many times have you ridden a horse? Do you like it?

*Child:* Yes, once.My mother taught me how to do it.

*Adult:* I rode one once. The horse got super nervous and started running. I never rode a horse again.

*Child:* **Well, there are also white horses.**

2.

*Adult:* What is this?

*Child:* Walnuts!

*Adult:* Do you like walnuts?

*Child:* I like them, but mom and dad like them for salads.

*Adult:* For the salads? Wow, they’re delicious. I also add walnuts to my salads, and sometimes peanuts, too.

*Child:* **Look, I caught a walnut!**

**Condition 4 – associated implicit none**

List A

1.

*Adult*: Were you looking forward to starting school, or would you have preferred to stay on vacation a bit longer?

*Child*: I was looking forward to it to be with my friends.

*Adult*: Of course. Didn’t you see them during the summer?

*Child*: No. Well, when summer ended, I saw my friend Aitor.

*Adult*: Ah, cool.

*Child*: **Do you know anyone named Aitor?**

2.

*Adult:* What do we need a first-aid kit for in the mountain?

*Child:* Because you can get hurt. I’ve never hurt myself.

*Adult:* No? Well, that’s lucky.

*Child:* Well, yes.

*Adult:* Okay, let’s put everything in the backpack. Let’s see if I can close the first-aid kit. Can you put the cookies in the backpack meanwhile?

*Child:* **Yeah, okay, let’s see. I ate cookies for breakfast not too long ago.**

List B

1.

*Adult:* You’ve never gone hiking?

*Child:* Yes, sometimes I have.

*Adult:* I really like going hiking.

*Child:* Yes?

*Adult:* Yes, I go hiking a lot. Do you know where Gorbea is?

*Child:* **Yes. I always go to the mountain by car.**

2.

*Adult:* Hey, did your friends come with you?

*Child:* Yes, because my father will invite them to eat some French fries when we finish.

*Adult:* Oh, why is that? Are you celebrating something?

*Child:* No, but he knows we love them.

*Adult:* He’s nice, isn’t he? What’s your father’s name?

*Child:* **Carlos. Was your name Sara?**

**Condition 5 – associated implicit question**

List A

1.

*Adult*: I think something is wrong with these animals. They look a bit sad.

*Child*: The little donkey has his head down. He’s crying.

*Adult*: Is he crying? Why?

*Child*: I don’t know.

*Adult*: Do you think his leg hurts?

*Child*: **The horse’s leg is already healed.**

2.

*Adult:* Let’s get to a place…

*Child:* There’re animals!

*Adult:* Wow, so many animals!

*Child:* Yeah!

*Adult:* You really liked animals, didn’t you?

*Child:* **This cow has a wound.**

List B

1.

*Adult:* When you go hiking, what do you carry in your backpack?

*Child:* Well, I don’t carry anything.

*Adult:* Nothing?

*Child:* I don’t carry a backpack.

*Adult:* You don’t carry a backpack when you go hiking?

*Child:* **My backpack is from Decathlon.**

2.

*Adult:* Do you know what I have at home?

*Child:* What?

*Adult:* I have a cat.

*Child:* What does it look like?

*Adult:* It’s gray. It has a lot of hair, it looks like a hairball. Do you know what Persian cats are like?

*Child:* **I have a friend named Toñi who has a cat. It’s orange and it has hair, too.**

**Condition 6 – associated implicit statement**

List A

1.

*Adult:* Come on, can you draw something back there?

*Child:* Yes.

*Adult:* Which do you prefer, markers or crayons?

*Child:* Markers.

*Adult:* Markers? Well, here you go. I’ll finish this drawing.

*Child:* **I have more markers than crayons at home.**

2.

*Adult:* Okay, and one last thing…

*Child:* The compass.

*Adult:* Very good. What’s the compass for?

*Child:* To tell us where the south and north are.

*Adult:* That’s right, and also the east and west. Okay, it’s very important so we don’t get lost.

*Child:* **My friend Joel has this watch with a compass. In gold color, with different drawings.**

List B

1.

*Adult:* Do you like drawing?

*Child:* Mmm, well…

*Adult:* More or less?

*Child:* Yes. What do I have to draw?

*Adult:* You have two choices. You can either finish a drawing that is already started or make a new one. Whatever you want.

*Child:* **People draw very well.**

2.

*Adult*: Have you ever planted anything?

*Child*: At school, we planted peas and other things.

*Adult*: I don’t like peas very much.

*Child*: Me neither.

*Adult*: My grandma has a vegetable garden, and she grows peppers and things like that. Then more grow, so she doesn’t have to buy them.

*Child*: **Our teacher has a vegetable garden and a mosquito bit her on the arm.**

**Condition 7 – topic reintroduction explicit none**

List A

1.

*Adult*: What’s your robin’s name?

*Child*: His name is, uh… I need to think.

*Adult*: Have you forgotten? It’s okay. Do you also have siblings or are you an only child?

*Child*: I’m an only child. I have cousins.

*Adult*: Alright, so your family is pretty big.

*Child*: Ah, yes. **Ah, I remember what he’s called now. He’s called Ruisi.**

2.

*Adult:* I don’t think that volcano will erupt, I don’t think so.

*Child:* Yes, it will erupt!

*Adult:* Look, let’s go see this farm now, okay? What animals are there on this farm?

*Child:* A horse, a donkey, a cow and a zebra.

*Adult:* I love zebras. Have you ever seen a zebra?

*Child:* **Yes. Do you know where the volcano is? Right next door.**

List B

1.

*Adult:* What’s your favorite animal?

*Child:* Cats and monkeys. I saw monkeys at the zoo.

*Adult:* Ah, how cool. Do you like dogs?

*Child:* Mmm, yes, I like them.

*Adult:* Ah, okay.

*Child:* **You know what? Have you ever been to the zoo?**

2.

*Adult:* Come on, let’s put all our things in the backpack before it gets dark. Come on, let’s go on a trip. Hey, what’s your favorite animal?

*Child:* Dogs.

*Adult:* Do you have any at home?

*Child:* No, because my mom is allergic.

*Adult:* She doesn’t want any, right?

*Child:* **Come on, come on, it’s already dark!**

**Condition 8 – topic reintroduction explicit question**

List A

1.

*Adult:* Ah, I don’t know that part of Galicia. I’ve been to Santiago once. Have you been there?

*Child:* Mmm, I don’t know.

*Adult:* It’s also in Galicia. Do you like going there to spend the summer with your grandmother?

*Child:* Yes. I like going to the pool.

*Adult:* Does she have a pool at her house?

*Child:* **And Pontevedra too.**

2.

*Adult:* Well, I’m good at painting, but I’m terrible at drawing like you’re doing. Have you drawn a tree?

*Child:* Yes.

*Adult:* Don’t suck your thumb. Do you want some paper?

*Child:* It itches. A mosquito bit me.

*Adult:* Oh, really? With the heat, mosquitoes are starting to come out… Hey, by the way, do you play any sports?

*Child:* **Wait, let me finish the tree.**

List B

1.

*Adult*: I have the gray marker. Do you want to borrow it?

*Child*: Yes, yes, yes.

*Adult*: Well, you told me you were with Julia too. Did you have fun with her?

*Child*: Yes, very much.

*Adult*: Hey, and these days when you don’t have school, what do you do at home?

*Child*: **Wait, just a bit more gray.**

2.

*Adult:* What do we need a compass for in the mountain?

*Child:* So we don’t get lost. I have one on my walking stick.

*Adult:* Your walking stick has a compass? Look, another thing we forgot: walking sticks.

*Child:* Yes. And I also usually bring sneakers, just in case. And an umbrella, too.

*Adult:* Ah, I have the umbrella here. What I haven’t brought are walking sticks, but never mind. Hey, it’s really cool that your walking sticks have a compass, isn’t it?

*Child:* **And a cap or sunglasses.**

**Condition 9 – topic reintroduction explicit statement**

List A

1.

*Adult:* Where do you usually go with your friends?

*Child:* Well, since you don’t know Vitoria… Although you might know my street. Do you know which one is the pedestrian street? I live there, in a building with eleven floors.

*Adult:* Yes, I know which one it is. Ah, I live on the seventh floor.

*Child:* I live on the fourth.

*Adult:* On the fourth, mhm. Come on, let’s draw a picture related to today’s trip.

*Child:* **Let’s see, ah, I also had to tell you about my friends.**

2.

*Adult:* Look at the backpack I’ve prepared. Let’s see what you think. What’s that?

*Child:* A first-aid kit.

*Adult:* In case we get hurt, right? Or in case there’s an accident. Have you ever had an accident on a trip?

*Child:* No, I don’t remember.

*Adult:* I did once. I remember going on a trip to a farm, we were riding bikes, and I fell off my bike down a mountain slope.

*Child:* **And this is a flashlight, right?**

List B

1.

*Adult:* You’re going to do great on the exam. Is it easy?

*Child:* Yes, it is. We’re practicing the prefixes ‘des’ and ‘in’, and the numeral determiners.

*Adult:* That’s cool, I love that. I studied linguistics. Do you know what that is?

*Child:* Yes, I do.

*Adult:* Well, I studied that. I’m a linguist.

*Child:* **Ah, and also the prefix ‘ir’.**

2.

*Adult*: What mountain have you been to?

*Child*: Well, it’s close to Landa.

*Adult*: Hey, and what do you usually take with you when you go hiking?

*Child*: Some food.

*Adult*: Ah, a sandwich, you told me earlier.

*Child*: **And I’ve been to a mountain in Barria**.

**Condition 10 – topic reintroduction implicit none**

List A

1.

*Adult*: What happened to this tree?

*Child*: All its leaves and walnuts have fallen because of autumn.

*Adult*: That’s right, leaves fall in autumn. Your birthday is in summer, right?

*Child*: Yes.

*Adult*: My birthday is in autumn, near Halloween.

*Child*: **I see. How should I plant the walnuts?**

2.

*Adult:* Come on, let’s go. Look, we’re in the mountain.

*Child:* There are fallen leaves.

*Adult:* Wow, there are lots of leaves on the ground.

*Child:* And that.

*Adult:* What’s that?

*Child:* **Chestnuts. Let’s put the leaves on the tree.**

List B

1.

*Adult:* Okay, what color do you want? I’ll take the brown one to finish off the dog. You’ll take the green one for the tree?

*Child:* Yes.

*Adult:* Did you say you do origami? What’s that?

*Child:* Well, folding…

*Adult:* Ah, okay, making shapes with…

*Child:* **Yes. I’d better take red.**

2.

*Adult:* What happened to this tree?

*Child:* All the leaves and walnuts fell off in autumn.

*Adult:* That’s right, in autumn all the leaves fall off. What’s your favorite season?

*Child:* Summer, because of the beach. I go with my family, and it’s on top of a mountain.

*Adult:* What? The beach?

*Child:* **Yes. Should I put the leaves?**

**Condition 11 – topic reintroduction implicit question**

List A

1.

*Adult:* Hey, do you do judo?

*Child:* Yes.

*Adult:* Ah, you do judo. How is it? Do you like it?

*Child:* No, I don’t like it.

*Adult:* What do you like doing?

*Child:* **There’s just fighting.**

2.

*Adult*: I live in Basauri, near Bilbao. Have you ever been to Bilbao?

*Child*: Yes, a few times. I’ve been to the beach, to the rocket…

*Adult*: The rocket?

*Child*: Yes.

*Adult*: What rocket?

*Child*: **Why is it called Basauri?**

List B

1.

*Adult:* Did you plant Lacasitos?

*Child:* Yes, one Lacasito.

*Adult:* Really? Wow, a Lacasitos tree would be great, wouldn’t it?

*Child:* Yes.

*Adult:* Well, we’ve already planted the walnuts, right?

*Child:* **If we plant a Lacasito, another one will grow, right?**

2.

*Adult:* What happened to this tree?

*Child:* Its leaves have fallen off.

*Adult:* Why? Because it’s…

*Child:* Autumn.

*Adult:* Autumn, very good, because it’s autumn. And how many leaves are there?

*Child:* **It’s the last day of autumn.**

**Condition 12 – topic reintroduction implicit statement**

List A

1.

*Adult*: What will grow if we plant a walnut?

*Child*: A walnut tree.

*Adult*: That’s right. Come on, help me plant them. Great job!

*Child*: Done.

*Adult*: Now what we’re going to do is check these animals out.

*Child*: **The walnuts are spoiled.**

2.

*Adult:* What an original drawing.

*Child:* Well, give me another piece of paper.

*Adult:* Anotehr piece of paper? Mmm I can think of something better. Let’s watch a video on my phone, okay? I’ll keep the drawing to put it up in my house, because I really like it. Can I?

*Child:* Yes.

*Adult:* I have a video you’re really going to like.

*Child:* **Do you have another piece of paper to draw on?**

List B

1.

*Adult:* Very good, the leaves have fallen because it’s autumn. And we can also see walnuts, right? What can we do with these walnuts?

*Child:* Pick them up.

*Adult:* Hey, do you know which animal really likes walnuts?

*Child:* I don’t know.

*Adult:* Squirrels. Squirrels love walnuts.

*Child:* **It’s cold in autumn.**

2.

*Adult:* Look, I have some pictures here. You can color the lion, the girl with the dog, the sharks…

*Child:* The sharks, then.

*Adult:* Okay.

*Child:* I love lions too.

*Adult:* Me too. Did you know I saw a lion last week? I saw it at the zoo.

*Child:* **What color are sharks? Gray?**

**Condition 13 – non-associated explicit none**

List A

1.

*Adult:* Look, you can choose between markers and crayons.

*Child:* I prefer a pencil.

*Adult:* Okay, so which pencil?

*Child:* A normal pencil.

*Adult:* Here you go. What are you drawing? Oh, it’s us.

*Child:* **Yes. Did you know that there are lots of zebras in the jungles of Africa?**

2.

*Adult:* There are animals that fly too.

*Child:* Oh, look, a bird.

*Adult:* Well, what’s your favorite animal?

*Child:* An owl.

*Adult:* Oh, an owl with giant eyes.

*Child:* **Yes, with giant eyes. Look, we can do this with the leaves.**

List B

1.

*Adult*: Did you go hiking to the Gorbea mountain?

*Child*: Yes.

*Adult*: How cool! Who did you go with?

*Child*: With many teachers.

*Adult*: Ah, with your school teachers? I thought you had gone with your family.

*Child*: **Yes. Hey, look, I have a video game called ‘spy camera’.**

2.

*Adult:* Ah, does your uncle have a kitten too?

*Child:* Yes.

*Adult:* Do you know what my cat looks like?

*Child:* What does it look like?

*Adult:* It’s gray and furry. It looks like a furry ball.

*Child:* **Yes, it does. Look at this walnut.**

**Condition 14 – non-associated explicit question**

List A

1.

*Adult*: Look what I have. What’s this?

*Child*: A compass. It shows you the north, south, east and west.

*Adult*: Do you know how to use it?

*Child*: Well, no, because I don’t know which direction my house is in.

*Adult*: Of course. Alright, let’s go. Do you want to carry the backpack?

*Child*: **Ah, one thing. Do you know one of the things that scares me the most? Heights.**

2.

*Adult:* Well, let’s go with your parents.

*Child:* Okay, and if I can, I’ll eat a Kinder.

*Adult:* I don’t have any Kinder, eh.

*Child:* I know, the Kinders are in the cafeteria.

*Adult:* Ah, you’ve already spotted them. Well, did you have a good time?

*Child:* **Hey, sorry, I have to take out the leaves.**

List B

1.

*Adult:* Have you ever broken a bone?

*Child:* No, but my brother has. He’s broken his arm twice.

*Adult:* Poor thing. Well, let’s get back to the activity. Is the zebra recovered?

*Child:* Yes.

*Adult:* Very good. Come on, let’s leave our animal friends here. We’ll say goodbye to them now, okay?

*Child:* **Look, you know what? There’s a video game called Minecraft. Do you know it?**

2.

*Adult:* What’s wrong with this tree?

*Child:* It has no leaves.

*Adult:* Why do you think it has no leaves?

*Child:* Because they’ve fallen off?

*Adult:* Very good, they’ve fallen off, haven’t they? And when do leaves fall from trees?

*Child:* **Wait, we’re lost!**

**Condition 15 – non-associated explicit statement**

List A

1.

*Adult:* Well, we have two things left to do.

*Child:* What are they?

*Adult:* Draw a picture…

*Child:* Can it be of animals?

*Adult:* It has to be something related to what we did today.

*Child:* **Oh, a nut has fallen off.**

2.

*Adult:* Hey, what’s your favorite season of the year? Which one do you like best?

*Child:* Summer.

*Adult:* Summer? Why?

*Child:* Because it’s very sunny.

*Adult:* I can’t stand the heat.

*Child:* **Look, look. Hello, sheep.**

List B

1.

*Adult:* What do you like doing in your free time?

*Child:* Play on the PlayStation.

*Adult:* What games do you have on the PlayStation?

*Child:* Among Us, el Minecraft…

*Adult:* Wow, I’ve never played Among Us.

*Child:* **Ooops, a target.**

2.

*Adult*: With the balls it’s harder to hit the target. Do you want to try with a dart?

*Child*: Yes.

*Adult*: Come a bit closer.

*Child*: Well, look, I hit the number ten!

*Adult*: Ten! Then I’ve won.

*Child*: **Look, a zebra.**

**Condition 16 – non-associated implicit none**

List A

1.

*Adult*: Well, I like spring more than summer.

*Child*: I don’t.

*Adult*: What do you usually do in the summer? Do you usually go on vacation?

*Child*: Mhm.

*Adult*: Yes?

*Child*: **Yes.** **I’m going to open the fridge.**

2.

*Adult:* What animals do you have?

*Child:* I have a cat and a dog.

*Adult:* Oh, I have a cat too.

*Child:* And a dog?

*Adult:* No, not a dog, a cat. I have a Persian cat. Do you know what breed that is?

*Child:* **Yes. Let’s play with that now.**

List B

1.

*Adult:* Don’t you know what walnuts look like inside?

*Child:* No.

*Adult:* Well, I don’t know how to explain it. My grandmother has some, so one day I’ll bring you a real one and show it to you.

*Child:* Yes.

*Adult:* Okay?

*Child:* **Yes. I’ve known you for a long time.**

2.

*Adult:* Let’s see where you can paint something.

*Child:* Who drew those pictures?

*Adult:* Other children.

*Child:* And they had fun, didn’t they?

*Adult:* Of course. Are you having fun?

*Child:* **Yes. Bird, what are you doing on my hand?**

**Condition 17 – non-associated implicit question**

List A

1.

*Adult:* Do you have a very big house?

*Child:* Uh, yes.

*Adult:* Hey, what did you take with you when you went camping?

*Child:* I took an ice cream.

*Adult:* Ice cream? I love ice cream. What flavor do you like?

*Child:* **Then I was hot during the dream, but when I woke up I was cold.**

2.

*Adult:* Come on, this will be our mountain for today. What do you think?

*Child:* Okay.

*Adult:* Who will carry the backpack? You or me?

*Child:* Me.

*Adult:* Dou you know any famous mountains? Can you tell me about any mountains you’ve been to?

*Child:* **What is this picture?**

List B

1.

*Adult:* What do you like more, going hiking or reading books?

*Child:* Reading books.

*Adult*: Ah, very good. Hey, by the way, do you know where your cap is from?

*Child*: From Catalonia?

*Adult*: No. It’s from Sopelana. Do you know where Sopelana is?

*Child:* **My teacher always lets me use the computer when I’ve done my work.**

2.

*Adult:* And do you see your grandparents’ dog a lot?

*Child:* Yes.

*Adult:* Does he get along with your cat or not?

*Child:* But my cat doesn’t go to my grandparents’ house.

*Adult:* Oh, he’s never been there. So they’ve never met?

*Child:* **Which roll of tape has more tape on it?**

**Condition 18 – non-associated implicit statement**

List A

1.

*Adult:* So, how are you feeling about going back to school? Are you looking forward to it or not?

*Child:* No.

*Adult:* No, right? What a drag.

*Child:* Yes.

*Adult:* Wake up early again, homework again…

*Child:* **Mom is working now.**

2.

*Adult:* Have you ever been on a trip to the mountains?

*Child:* No.

*Adult:* Well, we’re going on a trip to the mountains today. What do I have here?

*Child:* A backpack.

*Adult:* Okay, we’ll see what I have inside the backpack afterwards. Now you have to find four animals that are hidden around the room.

*Child:* **Is this walnut real?**

List B

1.

*Adult*: Okay, it’s autumn we’ve said, right?

*Child*: Yes.

*Adult*: Your birthday is in the summer, right?

*Child*: Yes.

*Adult*: My birthday is in autumn.

*Child*: **What do I do with this?**

2.

*Adult:* Indeed, how did you know that the compass was in English?

*Child:* Because it says ‘W’ here.

*Adult:* Do you know how to say ‘oeste’ in English?

*Child:* How?

*Adult:* West.

*Child:* **We have to open the walnuts.**

**3.4. Attention checks**

Each list included two attention checks to ensure participants were paying attention to the task. The attention checks consisted of multiple-choice questions about the content of the preceding conversation. For example:

What was the previous conversation about? Select the correct answer.

*A: About mountains.*

*B: About drawings.*

*C: About nuts.*

**4. Items pretest**

The following items, initially included in the pretest, were modified before the main experiment to ensure that raters evaluated the child’s utterance without being influenced by factors unrelated to conversation structure or topic shifts:

**Spanish:**

1.

*Adulto:* ¿Tú cuando vas al monte qué cosas te llevas en la mochila?

*Niño:* Pues no llevo nada.

*Adulto:* ¿Nada?

*Niño:* No llevo mochila.

*Adulto:* ¿No llevas mochila al monte?

*Niño:* **Mi mochila es de ~~Ternua~~ Decathlon.**

2.

*Adulto:* Vas a hacer genial el examen. ¿Es fácil?

*Niño:* Pues sí. Estamos ensayando los prefijos ‘des’ e ‘in’ y los determinantes numerales.

*Adulto:* Qué guay, a mí me encanta. Yo estudié lingüística. ¿Sabes lo que es?

*Niño:* Sí, lo sé.

*Adulto:* Pues yo estudié eso. Yo soy lingüista.

*Niño:* **Ah, y también ~~la diéresis~~ el prefijo ‘ir’.**

3.

*Adulto:* Venga, ¿dibujas ahí detrás algo?

*Niño:* Sí.

*Adulto:* ¿Qué prefieres, rotus o pinturas?

*Niño:* Rotus.

*Adulto:* ¿Rotus? Pues coge. Yo voy a terminar este dibujo.

*Niño:* **~~Los rotuladores se inventaron más tarde que las pinturas.~~ Tengo más rotus que pinturas en casa.**

**English:**

1.

*Adult:* When you go hiking, what do you carry in your backpack?

*Child:* Well, I don’t carry anything.

*Adult:* Nothing?

*Child:* I don’t carry a backpack.

*Adult:* You don’t carry a backpack when you go hiking?

*Child:* **My backpack is from ~~Ternua~~ Decathlon.**

*(“Ternua” was replaced with “Decathlon” after a participant in the pretest indicated that the Ternua brand may only be familiar in some regions of Spain)*

2.

*Adult:* You’re going to do great on the exam. Is it easy?

*Child:* Yes, it is. We’re practicing the prefixes ‘des’ and ‘in’, and the numeral determiners.

*Adult:* That’s cool, I love that. I studied linguistics. Do you know what that is?

*Child:* Yes, I do.

*Adult:* Well, I studied that. I’m a linguist.

*Child:* **Ah, and also the ~~diaeresis~~ prefix ‘ir’.**

*(“Diaeresis” was replaced with “prefix ‘ir’” since the former might sound too pedantic for a child)*

3.

*Adult:* Come on, can you draw something back there?

*Child:* Yes.

*Adult:* Which do you prefer, markers or crayons?

*Child:* Markers.

*Adult:* Markers? Well, here you go. I’ll finish this drawing.

*Child:* **~~Markers were invented after crayons.~~ I have more markers than crayons at home.**

*(Raters might think that a child of this age is unlikely to know that markers were invented after crayons. We modified the utterance to “I have more markers than crayons at home”, which is still associated with the immediate previous topic in the conversation)*

**References**

Zehr, J., & Schwarz, F. (2018). PennController for Internet Based Experiments (IBEX). <https://doi.org/10.17605/OSF.IO/MD832>
