# Supplementary material for: Conversational Topic Shifts and Topic Maintenance in Autistic and Neurotypical Children
Source: Autism Res. 2026 Feb 18;19(4):e70204. doi: 10.1002/aur.70204 (PMC13087834; doi:10.1002/aur.70204)
Supplement: Supplementary file 1 — Data S1: Supporting Information. [file AUR-19-0-s001.zip › Supplementary Materials/Supplementary Material 2_Rating task/Commented R code 2.html]

Naturalness judgments of topic shifts


# Naturalness judgments of topic shifts

#### 2025-02-19

Load necessary packages.

```
library(tidyverse)
```

```
## -- Attaching core tidyverse packages ------------------------ tidyverse 2.0.0 --
## v dplyr     1.1.4     v readr     2.1.5
## v forcats   1.0.0     v stringr   1.5.1
## v ggplot2   3.5.1     v tibble    3.2.1
## v lubridate 1.9.4     v tidyr     1.3.1
## v purrr     1.0.2     
## -- Conflicts ------------------------------------------ tidyverse_conflicts() --
## x dplyr::filter() masks stats::filter()
## x dplyr::lag()    masks stats::lag()
## i Use the conflicted package (<http://conflicted.r-lib.org/>) to force all conflicts to become errors
```

```
library(dplyr)
library(readxl)
library(ggplot2)
library(ggeffects)
library(emmeans)
```

```
## Welcome to emmeans.
## Caution: You lose important information if you filter this package's results.
## See '? untidy'
```

```
library(ordinal)
```

```
## 
## Adjuntando el paquete: 'ordinal'
## 
## The following object is masked from 'package:dplyr':
## 
##     slice
```

```
library(cluster)
library(ltm)
```

```
## Cargando paquete requerido: MASS
## 
## Adjuntando el paquete: 'MASS'
## 
## The following object is masked from 'package:dplyr':
## 
##     select
## 
## Cargando paquete requerido: msm
## Cargando paquete requerido: polycor
```

```
library(psych)
```

```
## 
## Adjuntando el paquete: 'psych'
## 
## The following object is masked from 'package:ltm':
## 
##     factor.scores
## 
## The following object is masked from 'package:polycor':
## 
##     polyserial
## 
## The following objects are masked from 'package:ggplot2':
## 
##     %+%, alpha
```

# Pretest

```
# load dataset

results_pretest <- read_excel("pretest.xlsx") 
View(results_pretest)

# assess the internal consistency of the critical items (Cronbach's alpha)

selected_columns <- dplyr::select(results_pretest,condition, choice)
glimpse(selected_columns)
```

```
## Rows: 1,040
## Columns: 2
## $ condition <chr> "13", "16", "9", "2", "5", "4", "6", "7", "12", "17", "14", ~
## $ choice    <dbl> 5, 1, 2, 4, 5, 7, 6, 5, 6, 2, 2, 3, 3, 7, 3, 3, 5, 5, 7, 5, ~
```

```
selected_col_filtered <- selected_columns %>% 
  filter(condition != 19)

wide_data <- selected_col_filtered %>% # transform the data into wide format 
  group_by(condition) %>%
  mutate(row = row_number()) %>%  
  ungroup() %>%
  pivot_wider(names_from = row, values_from = choice, names_prefix = "choice_")

cronbach.alpha(wide_data)
```

```
## 
## Cronbach's alpha for the 'wide_data' data-set
## 
## Items: 53
## Sample units: 18
## alpha: 0.926
```

# Main experiment

1. Load the dataset, define the relevant variables and extract the
   relevant descriptive statistics.

```
# load dataset

judgments_final <- read_excel("main_experiment.xlsx") 
View(judgments_final)

# define variables

judgments_final$participant <- as.factor(judgments_final$participant)
judgments_final$age <- as.numeric(judgments_final$age)
judgments_final$gender <- as.factor(judgments_final$gender)
judgments_final$choice <- as.numeric(judgments_final$choice)
judgments_final$list <- as.factor(judgments_final$list)
judgments_final$condition <- factor(judgments_final$condition, levels = 1:19)
judgments_final$condition_name <- as.factor(judgments_final$condition_name)
View(judgments_final)

# descriptive statistics of participants' characteristics

list_part <- judgments_final %>% # 48 participants in list A, 48 in list B
  group_by(list) %>%
  summarise(
   n = n_distinct(participant))

gender_part <- judgments_final %>% # 38 females, 57 males, 1 non-binary
  group_by(gender) %>%
  summarise(
   n = n_distinct(participant))

mean_age <- judgments_final %>%
  summarise(mean_age = mean(age, na.rm = TRUE),  SD = round(sd(age, na.rm = TRUE),2)) # (M = 35.5, SD = 9.87)


# average ratings per condition (type of topic shift)

average_ratings <- judgments_final %>%
    group_by(condition) %>%
    summarise(average_score = mean(choice))

average_ratings
```

2. Perform statistical analyses. Ratings were analyzed with a
   cumulative link mixed model with condition included as fixed effect and
   participant and item as random effects.

```
# cumulative link mixed model 

judgments_final$choice <- as.factor(judgments_final$choice)

clmm_model <- clmm(choice ~ condition + (1|participant) + (1|item), data = judgments_final)
summary(clmm_model)
```

```
## Cumulative Link Mixed Model fitted with the Laplace approximation
## 
## formula: choice ~ condition + (1 | participant) + (1 | item)
## data:    judgments_final
## 
##  link  threshold nobs logLik   AIC      niter       max.grad cond.H 
##  logit flexible  3840 -6086.10 12224.20 5030(20124) 1.38e-03 1.1e+04
## 
## Random effects:
##  Groups      Name        Variance Std.Dev.
##  participant (Intercept) 0.6413   0.8008  
##  item        (Intercept) 0.2728   0.5223  
## Number of groups:  participant 96,  item 40 
## 
## Coefficients:
##             Estimate Std. Error z value Pr(>|z|)    
## condition2   -1.7996     0.5569  -3.231 0.001232 ** 
## condition3   -1.3826     0.5562  -2.486 0.012920 *  
## condition4   -0.4744     0.5571  -0.852 0.394487    
## condition5   -2.0422     0.5554  -3.677 0.000236 ***
## condition6   -0.6798     0.5571  -1.220 0.222368    
## condition7   -0.9698     0.5564  -1.743 0.081336 .  
## condition8   -2.0502     0.5565  -3.684 0.000230 ***
## condition9   -1.3963     0.5566  -2.509 0.012116 *  
## condition10  -1.5202     0.5568  -2.730 0.006326 ** 
## condition11  -2.0324     0.5573  -3.647 0.000265 ***
## condition12  -1.7255     0.5558  -3.105 0.001905 ** 
## condition13  -2.1393     0.5556  -3.850 0.000118 ***
## condition14  -3.3279     0.5579  -5.965 2.45e-09 ***
## condition15  -4.0941     0.5608  -7.301 2.86e-13 ***
## condition16  -3.4566     0.5591  -6.182 6.31e-10 ***
## condition17  -4.8125     0.5644  -8.527  < 2e-16 ***
## condition18  -4.0084     0.5602  -7.155 8.38e-13 ***
## condition19   1.6265     0.4914   3.310 0.000933 ***
## ---
## Signif. codes:  0 '***' 0.001 '**' 0.01 '*' 0.05 '.' 0.1 ' ' 1
## 
## Threshold coefficients:
##     Estimate Std. Error z value
## 1|2  -4.2618     0.4094 -10.409
## 2|3  -3.3762     0.4075  -8.285
## 3|4  -2.6521     0.4063  -6.528
## 4|5  -1.9598     0.4053  -4.836
## 5|6  -1.0110     0.4042  -2.501
## 6|7   0.2130     0.4038   0.528
```

Model predictions were extracted using ggeffect.

```
# extract model predictions

predictions <- ggeffect(clmm_model, terms = "condition")
```

```
## You are calculating adjusted predictions on the population-level (i.e.
##   `type = "fixed"`) for a *generalized* linear mixed model.
##   This may produce biased estimates due to Jensen's inequality. Consider
##   setting `bias_correction = TRUE` to correct for this bias.
##   See also the documentation of the `bias_correction` argument.
```

```
# extract the mean predicted ratings per condition

df_predictions <- as.data.frame(predictions)

predicted_ratings <- df_predictions %>%
  mutate(response.level = as.numeric(gsub("X", "", response.level))) %>%
  group_by(x) %>%
  summarize(mean_score = sum(response.level * predicted, na.rm = TRUE),  sd_score = sd(response.level * predicted, na.rm = TRUE), ci_low_mean = sum(response.level * conf.low),
    ci_high_mean = sum(response.level * conf.high)
  )
predicted_ratings <- predicted_ratings %>%
  rename(condition = x)

predicted_ratings
```

```
# plot the distribution of ratings with fitted values

judgments_final$condition <- factor(judgments_final$condition, levels = as.character(1:19))
grouped_colors <- c(
  # Assign different colors to distinguish topic shift types
   "#B05285",  "#E285BF",  "#F2A6C9",  "#C95A8D",  "#E285BF",  "#F2A6C9", "#3399CC",  "#66B2E6", "#99CCFF",  "#3399CC", "#66B2E6", "#99CCFF",
    "#7DCA3E", "#A6D854", "#B0D86B", "#7DCA3E", "#A6D854","#B0D86B", "#FF6F00", 
  "#FF69B4" )

judgments_final$choice <- as.numeric(judgments_final$choice)

plot_judgments <- ggplot(judgments_final, aes(x = condition, y = choice, fill = condition)) +
  geom_boxplot(aes(color = condition),
               fill = "white",        
               alpha = 0.6, 
                 size =0.8,
               outlier.color = "brown",
               show.legend = FALSE) +
  geom_point(data = predicted_ratings, 
             aes(x = condition, y = mean_score, color = condition), 
             size = 6, shape = 16, show.legend = FALSE) +
  scale_y_continuous(expand = c(0, 0.05), breaks = seq(0, 7, by = 1)) +
  scale_fill_manual(values = grouped_colors) +  
  scale_color_manual(values = grouped_colors) +  
  labs(
    title = "",
    x = "Condition",
    y = "Score"
  ) +
  theme_bw() +
  theme(
    axis.text.x = element_text(angle = 45, hjust = 1), 
    plot.title = element_text(hjust = 0.5)
  )

plot_judgments
```

To assess which conditions differed significantly, post-hoc (emmeans)
analyses were carried out.

```
# post-hoc analyses

emmeans_clmm <- emmeans(clmm_model, ~ condition)
emmeans_df <- as.data.frame(emmeans_clmm)

options(max.print = 10000)
pairwise_clmm <- pairs(emmeans_clmm, adjust = "tukey") # pairwise comparisons

pairwise_clmm
```

```
##  contrast                  estimate    SE  df z.ratio p.value
##  condition1 - condition2    1.79961 0.557 Inf   3.231  0.1185
##  condition1 - condition3    1.38261 0.556 Inf   2.486  0.5687
##  condition1 - condition4    0.47437 0.557 Inf   0.852  1.0000
##  condition1 - condition5    2.04220 0.555 Inf   3.677  0.0292
##  condition1 - condition6    0.67981 0.557 Inf   1.220  0.9995
##  condition1 - condition7    0.96979 0.556 Inf   1.743  0.9666
##  condition1 - condition8    2.05022 0.557 Inf   3.684  0.0285
##  condition1 - condition9    1.39627 0.557 Inf   2.509  0.5508
##  condition1 - condition10   1.52018 0.557 Inf   2.730  0.3824
##  condition1 - condition11   2.03242 0.557 Inf   3.647  0.0324
##  condition1 - condition12   1.72551 0.556 Inf   3.105  0.1664
##  condition1 - condition13   2.13925 0.556 Inf   3.850  0.0156
##  condition1 - condition14   3.32790 0.558 Inf   5.965  <.0001
##  condition1 - condition15   4.09412 0.561 Inf   7.301  <.0001
##  condition1 - condition16   3.45661 0.559 Inf   6.182  <.0001
##  condition1 - condition17   4.81252 0.564 Inf   8.527  <.0001
##  condition1 - condition18   4.00839 0.560 Inf   7.155  <.0001
##  condition1 - condition19  -1.62652 0.491 Inf  -3.310  0.0947
##  condition2 - condition3   -0.41700 0.554 Inf  -0.753  1.0000
##  condition2 - condition4   -1.32524 0.555 Inf  -2.387  0.6455
##  condition2 - condition5    0.24259 0.552 Inf   0.439  1.0000
##  condition2 - condition6   -1.11979 0.555 Inf  -2.018  0.8798
##  condition2 - condition7   -0.82982 0.554 Inf  -1.497  0.9934
##  condition2 - condition8    0.25061 0.553 Inf   0.453  1.0000
##  condition2 - condition9   -0.40334 0.554 Inf  -0.728  1.0000
##  condition2 - condition10  -0.27943 0.554 Inf  -0.504  1.0000
##  condition2 - condition11   0.23281 0.554 Inf   0.420  1.0000
##  condition2 - condition12  -0.07410 0.553 Inf  -0.134  1.0000
##  condition2 - condition13   0.33964 0.552 Inf   0.615  1.0000
##  condition2 - condition14   1.52829 0.554 Inf   2.758  0.3627
##  condition2 - condition15   2.29452 0.557 Inf   4.122  0.0054
##  condition2 - condition16   1.65700 0.555 Inf   2.985  0.2235
##  condition2 - condition17   3.01292 0.560 Inf   5.381  <.0001
##  condition2 - condition18   2.20878 0.556 Inf   3.972  0.0098
##  condition2 - condition19  -3.42613 0.491 Inf  -6.985  <.0001
##  condition3 - condition4   -0.90824 0.555 Inf  -1.638  0.9822
##  condition3 - condition5    0.65959 0.552 Inf   1.195  0.9996
##  condition3 - condition6   -0.70279 0.554 Inf  -1.268  0.9992
##  condition3 - condition7   -0.41282 0.554 Inf  -0.746  1.0000
##  condition3 - condition8    0.66761 0.553 Inf   1.207  0.9996
##  condition3 - condition9    0.01366 0.553 Inf   0.025  1.0000
##  condition3 - condition10   0.13757 0.554 Inf   0.249  1.0000
##  condition3 - condition11   0.64982 0.554 Inf   1.173  0.9997
##  condition3 - condition12   0.34290 0.552 Inf   0.621  1.0000
##  condition3 - condition13   0.75665 0.552 Inf   1.371  0.9977
##  condition3 - condition14   1.94529 0.554 Inf   3.512  0.0509
##  condition3 - condition15   2.71152 0.557 Inf   4.872  0.0002
##  condition3 - condition16   2.07400 0.555 Inf   3.737  0.0237
##  condition3 - condition17   3.42992 0.560 Inf   6.125  <.0001
##  condition3 - condition18   2.62578 0.556 Inf   4.722  0.0004
##  condition3 - condition19  -3.00913 0.490 Inf  -6.147  <.0001
##  condition4 - condition5    1.56783 0.554 Inf   2.832  0.3132
##  condition4 - condition6    0.20545 0.556 Inf   0.370  1.0000
##  condition4 - condition7    0.49542 0.555 Inf   0.893  1.0000
##  condition4 - condition8    1.57585 0.555 Inf   2.840  0.3077
##  condition4 - condition9    0.92190 0.555 Inf   1.661  0.9794
##  condition4 - condition10   1.04581 0.555 Inf   1.884  0.9315
##  condition4 - condition11   1.55806 0.556 Inf   2.804  0.3313
##  condition4 - condition12   1.25114 0.554 Inf   2.258  0.7397
##  condition4 - condition13   1.66489 0.554 Inf   3.006  0.2125
##  condition4 - condition14   2.85353 0.556 Inf   5.132  <.0001
##  condition4 - condition15   3.61976 0.559 Inf   6.477  <.0001
##  condition4 - condition16   2.98224 0.557 Inf   5.352  <.0001
##  condition4 - condition17   4.33816 0.562 Inf   7.714  <.0001
##  condition4 - condition18   3.53402 0.558 Inf   6.330  <.0001
##  condition4 - condition19  -2.10089 0.490 Inf  -4.287  0.0027
##  condition5 - condition6   -1.36238 0.553 Inf  -2.462  0.5877
##  condition5 - condition7   -1.07241 0.553 Inf  -1.941  0.9118
##  condition5 - condition8    0.00802 0.552 Inf   0.015  1.0000
##  condition5 - condition9   -0.64593 0.552 Inf  -1.169  0.9997
##  condition5 - condition10  -0.52202 0.552 Inf  -0.945  1.0000
##  condition5 - condition11  -0.00977 0.553 Inf  -0.018  1.0000
##  condition5 - condition12  -0.31669 0.551 Inf  -0.575  1.0000
##  condition5 - condition13   0.09706 0.551 Inf   0.176  1.0000
##  condition5 - condition14   1.28570 0.552 Inf   2.328  0.6900
##  condition5 - condition15   2.05193 0.555 Inf   3.698  0.0271
##  condition5 - condition16   1.41441 0.553 Inf   2.556  0.5139
##  condition5 - condition17   2.77033 0.558 Inf   4.963  0.0001
##  condition5 - condition18   1.96619 0.554 Inf   3.547  0.0453
##  condition5 - condition19  -3.66872 0.489 Inf  -7.503  <.0001
##  condition6 - condition7    0.28997 0.555 Inf   0.523  1.0000
##  condition6 - condition8    1.37040 0.555 Inf   2.471  0.5804
##  condition6 - condition9    0.71645 0.555 Inf   1.291  0.9989
##  condition6 - condition10   0.84036 0.555 Inf   1.514  0.9925
##  condition6 - condition11   1.35261 0.555 Inf   2.435  0.6081
##  condition6 - condition12   1.04569 0.554 Inf   1.888  0.9302
##  condition6 - condition13   1.45944 0.554 Inf   2.636  0.4521
##  condition6 - condition14   2.64809 0.556 Inf   4.765  0.0003
##  condition6 - condition15   3.41431 0.559 Inf   6.113  <.0001
##  condition6 - condition16   2.77679 0.557 Inf   4.986  0.0001
##  condition6 - condition17   4.13271 0.562 Inf   7.353  <.0001
##  condition6 - condition18   3.32857 0.558 Inf   5.965  <.0001
##  condition6 - condition19  -2.30634 0.490 Inf  -4.705  0.0004
##  condition7 - condition8    1.08043 0.554 Inf   1.951  0.9079
##  condition7 - condition9    0.42648 0.554 Inf   0.770  1.0000
##  condition7 - condition10   0.55039 0.554 Inf   0.993  1.0000
##  condition7 - condition11   1.06264 0.555 Inf   1.916  0.9207
##  condition7 - condition12   0.75572 0.553 Inf   1.366  0.9978
##  condition7 - condition13   1.16946 0.553 Inf   2.116  0.8295
##  condition7 - condition14   2.35811 0.555 Inf   4.250  0.0032
##  condition7 - condition15   3.12433 0.558 Inf   5.604  <.0001
##  condition7 - condition16   2.48682 0.556 Inf   4.473  0.0012
##  condition7 - condition17   3.84274 0.561 Inf   6.850  <.0001
##  condition7 - condition18   3.03860 0.557 Inf   5.456  <.0001
##  condition7 - condition19  -2.59631 0.490 Inf  -5.303  <.0001
##  condition8 - condition9   -0.65395 0.553 Inf  -1.182  0.9997
##  condition8 - condition10  -0.53004 0.554 Inf  -0.957  1.0000
##  condition8 - condition11  -0.01779 0.554 Inf  -0.032  1.0000
##  condition8 - condition12  -0.32471 0.552 Inf  -0.588  1.0000
##  condition8 - condition13   0.08904 0.552 Inf   0.161  1.0000
##  condition8 - condition14   1.27768 0.553 Inf   2.308  0.7041
##  condition8 - condition15   2.04391 0.556 Inf   3.676  0.0293
##  condition8 - condition16   1.40639 0.555 Inf   2.536  0.5295
##  condition8 - condition17   2.76231 0.559 Inf   4.939  0.0001
##  condition8 - condition18   1.95817 0.555 Inf   3.525  0.0487
##  condition8 - condition19  -3.67674 0.490 Inf  -7.500  <.0001
##  condition9 - condition10   0.12391 0.554 Inf   0.224  1.0000
##  condition9 - condition11   0.63616 0.554 Inf   1.148  0.9998
##  condition9 - condition12   0.32924 0.553 Inf   0.595  1.0000
##  condition9 - condition13   0.74299 0.553 Inf   1.345  0.9982
##  condition9 - condition14   1.93163 0.554 Inf   3.484  0.0556
##  condition9 - condition15   2.69786 0.557 Inf   4.843  0.0002
##  condition9 - condition16   2.06034 0.556 Inf   3.709  0.0261
##  condition9 - condition17   3.41626 0.560 Inf   6.096  <.0001
##  condition9 - condition18   2.61212 0.556 Inf   4.694  0.0004
##  condition9 - condition19  -3.02279 0.490 Inf  -6.169  <.0001
##  condition10 - condition11  0.51225 0.554 Inf   0.924  1.0000
##  condition10 - condition12  0.20533 0.553 Inf   0.371  1.0000
##  condition10 - condition13  0.61908 0.553 Inf   1.120  0.9998
##  condition10 - condition14  1.80772 0.554 Inf   3.260  0.1092
##  condition10 - condition15  2.57395 0.557 Inf   4.621  0.0006
##  condition10 - condition16  1.93643 0.556 Inf   3.486  0.0553
##  condition10 - condition17  3.29235 0.560 Inf   5.875  <.0001
##  condition10 - condition18  2.48821 0.557 Inf   4.471  0.0012
##  condition10 - condition19 -3.14670 0.490 Inf  -6.418  <.0001
##  condition11 - condition12 -0.30692 0.553 Inf  -0.555  1.0000
##  condition11 - condition13  0.10683 0.553 Inf   0.193  1.0000
##  condition11 - condition14  1.29548 0.554 Inf   2.337  0.6831
##  condition11 - condition15  2.06170 0.557 Inf   3.703  0.0267
##  condition11 - condition16  1.42418 0.555 Inf   2.564  0.5073
##  condition11 - condition17  2.78010 0.560 Inf   4.964  0.0001
##  condition11 - condition18  1.97596 0.556 Inf   3.552  0.0446
##  condition11 - condition19 -3.65895 0.491 Inf  -7.453  <.0001
##  condition12 - condition13  0.41375 0.551 Inf   0.750  1.0000
##  condition12 - condition14  1.60239 0.553 Inf   2.897  0.2725
##  condition12 - condition15  2.36862 0.556 Inf   4.263  0.0030
##  condition12 - condition16  1.73110 0.554 Inf   3.124  0.1583
##  condition12 - condition17  3.08702 0.559 Inf   5.523  <.0001
##  condition12 - condition18  2.28288 0.555 Inf   4.113  0.0056
##  condition12 - condition19 -3.35203 0.489 Inf  -6.852  <.0001
##  condition13 - condition14  1.18865 0.552 Inf   2.152  0.8086
##  condition13 - condition15  1.95487 0.555 Inf   3.523  0.0491
##  condition13 - condition16  1.31735 0.553 Inf   2.380  0.6508
##  condition13 - condition17  2.67327 0.558 Inf   4.789  0.0003
##  condition13 - condition18  1.86913 0.554 Inf   3.372  0.0788
##  condition13 - condition19 -3.76578 0.489 Inf  -7.697  <.0001
##  condition14 - condition15  0.76622 0.555 Inf   1.379  0.9975
##  condition14 - condition16  0.12871 0.554 Inf   0.232  1.0000
##  condition14 - condition17  1.48462 0.558 Inf   2.658  0.4353
##  condition14 - condition18  0.68049 0.555 Inf   1.226  0.9995
##  condition14 - condition19 -4.95442 0.492 Inf -10.065  <.0001
##  condition15 - condition16 -0.63752 0.556 Inf  -1.146  0.9998
##  condition15 - condition17  0.71840 0.560 Inf   1.283  0.9990
##  condition15 - condition18 -0.08574 0.557 Inf  -0.154  1.0000
##  condition15 - condition19 -5.72065 0.496 Inf -11.543  <.0001
##  condition16 - condition17  1.35592 0.559 Inf   2.424  0.6172
##  condition16 - condition18  0.55178 0.556 Inf   0.992  1.0000
##  condition16 - condition19 -5.08313 0.494 Inf -10.299  <.0001
##  condition17 - condition18 -0.80414 0.560 Inf  -1.437  0.9960
##  condition17 - condition19 -6.43905 0.500 Inf -12.882  <.0001
##  condition18 - condition19 -5.63491 0.495 Inf -11.384  <.0001
## 
## P value adjustment: tukey method for comparing a family of 19 estimates
```

```
# plot emmeans results

plot_emmeans <- ggplot(emmeans_df, aes(x = factor(condition), y = emmean, color = factor(condition))) +
  geom_point(size = 5, show.legend = FALSE) +
  geom_errorbar(aes(ymin = asymp.LCL, ymax = asymp.UCL), 
                width = 0.2, size = 1, show.legend = FALSE) +  
  labs(x = "Condition", y = "Estimated Marginal Mean") +
  scale_color_manual(values = grouped_colors) +
  theme_bw() +
  theme(axis.text.x = element_text(angle = 90, hjust = 1))
```

```
## Warning: Using `size` aesthetic for lines was deprecated in ggplot2 3.4.0.
## i Please use `linewidth` instead.
## This warning is displayed once every 8 hours.
## Call `lifecycle::last_lifecycle_warnings()` to see where this warning was
## generated.
```

```
plot_emmeans
```

Since pairwise comparisons alone made it difficult to group
conditions together, a cluster analysis was performed to explore
potential groupings among the conditions based on their rating patterns,
providing a clearer understanding of how the different topic shift might
be related to each other in terms of perceived naturalness.

```
# clustering analysis (k-means)

# determine the optimal number of clusters using the Elbow method

clustering <- sapply(1:7, function(k) {
  kmeans_result <- kmeans(predicted_ratings$mean_score, centers = k, nstart = 25)
  sum(kmeans_result$withinss)
})

elbow_plot <- plot(1:7, clustering, type = "b", pch = 19, xlab = "Number of clusters", 
     ylab = "WSS", 
     main = "")
```

```
# run the k means with the three clusters 

kmeans_result_3 <- kmeans(predicted_ratings$mean_score, centers = 3, nstart = 25)
print(kmeans_result_3$centers)
```

```
##       [,1]
## 1 4.429442
## 2 2.382906
## 3 5.775540
```

```
data_clusters_3 <- predicted_ratings %>%
  mutate(cluster = kmeans_result_3$cluster) # assign the cluster number to each condition

# visualization

plot_cond_cl <- ggplot(data_clusters_3, aes(x = mean_score, y = condition, color = as.factor(cluster))) +
  geom_point(size = 5) +
    geom_text(aes(label = condition), vjust = -0.8, size = 4) +
  labs(title = "",
       x = "Mean predicted rating", y = "Condition",
       color = "Cluster") +
  theme_minimal()

# mean ratings per cluster 

table_clusters_3 <- data_clusters_3 %>%
  group_by(cluster) %>%
  summarise(
    mean_rating = mean(mean_score, na.rm = TRUE),
    sd_rating = sd(mean_score, na.rm = TRUE)
  )

# examine whether the clusters are statistically significant from each other

anova_result <- aov(mean_score ~ as.factor(cluster), data = data_clusters_3)
summary(anova_result)
```

```
##                    Df Sum Sq Mean Sq F value  Pr(>F)    
## as.factor(cluster)  2 29.356  14.678   81.98 3.9e-09 ***
## Residuals          16  2.865   0.179                    
## ---
## Signif. codes:  0 '***' 0.001 '**' 0.01 '*' 0.05 '.' 0.1 ' ' 1
```

```
TukeyHSD(anova_result)
```

```
##   Tukey multiple comparisons of means
##     95% family-wise confidence level
## 
## Fit: aov(formula = mean_score ~ as.factor(cluster), data = data_clusters_3)
## 
## $`as.factor(cluster)`
##          diff        lwr       upr    p adj
## 2-1 -2.046536 -2.6555212 -1.437550 6.00e-07
## 3-1  1.346098  0.7371126  1.955083 9.18e-05
## 3-2  3.392634  2.7021093  4.083158 0.00e+00
```

```
plot_clusters <- ggplot(data_clusters_3, aes(x = as.factor(cluster), y = mean_score, fill = cluster)) +
  geom_boxplot(show.legend = FALSE) +
  labs(title = "Average ratings per cluster", x = "Cluster", y = "Mean ratings") +
  theme_bw()

plot_clusters
```

```
# For the paper, cluster numbers were reassigned to make the interpretation more intuitive. The cluster with the lowest ratings (originally labeled as 2) is  labeled as 1 in the paper. The cluster originally labeled as 1 is labeled as 2, and the cluster 3 remains unchanged.
```
