# Supplementary material for: Conversational Topic Shifts and Topic Maintenance in Autistic and Neurotypical Children
Source: Autism Res. 2026 Feb 18;19(4):e70204. doi: 10.1002/aur.70204 (PMC13087834; doi:10.1002/aur.70204)
Supplement: Supplementary file 1 — Data S1: Supporting Information. [file AUR-19-0-s001.zip › Supplementary Materials/Supplementary Material 1_Group comparisons/Commented R code 1.html]

Comparison between autistic and neurotypical children


# Comparison between autistic and neurotypical children

#### 2025-02-20

Load necessary packages.

```
library(tidyverse)
```

```
## -- Attaching core tidyverse packages ------------------------ tidyverse 2.0.0 --
## v dplyr     1.1.4     v readr     2.1.5
## v forcats   1.0.0     v stringr   1.5.1
## v ggplot2   3.5.1     v tibble    3.2.1
## v lubridate 1.9.4     v tidyr     1.3.1
## v purrr     1.0.2     
## -- Conflicts ------------------------------------------ tidyverse_conflicts() --
## x dplyr::filter() masks stats::filter()
## x dplyr::lag()    masks stats::lag()
## i Use the conflicted package (<http://conflicted.r-lib.org/>) to force all conflicts to become errors
```

```
library(dplyr)
library(readxl)
library(ggplot2)
library(geomtextpath)
library(ggtext)
library(ggeffects)
library(emmeans)
```

```
## Welcome to emmeans.
## Caution: You lose important information if you filter this package's results.
## See '? untidy'
```

```
library(MASS)
```

```
## 
## Adjuntando el paquete: 'MASS'
## 
## The following object is masked from 'package:dplyr':
## 
##     select
```

```
library(pscl)
```

```
## Classes and Methods for R originally developed in the
## Political Science Computational Laboratory
## Department of Political Science
## Stanford University (2002-2015),
## by and under the direction of Simon Jackman.
## hurdle and zeroinfl functions by Achim Zeileis.
```

```
library(lme4)
```

```
## Cargando paquete requerido: Matrix
## 
## Adjuntando el paquete: 'Matrix'
## 
## The following objects are masked from 'package:tidyr':
## 
##     expand, pack, unpack
```

```
library(lmerTest)
```

```
## 
## Adjuntando el paquete: 'lmerTest'
## 
## The following object is masked from 'package:lme4':
## 
##     lmer
## 
## The following object is masked from 'package:stats':
## 
##     step
```

Load the dataset, define the relevant variables and extract the
relevant descriptive statistics of participants’ characteristics.

```
# load datasets

data_autistic <- read_excel("raw_asd.xlsx") 
data_neurotypical <- read_excel("raw_td.xlsx")

# merge datasets

data_groups <- rbind(data_autistic, data_neurotypical)
View(data_groups)

# define variables

data_groups$group <- as.factor(data_groups$group)
data_groups$age_ca_months <- as.numeric(data_groups$age_ca_months)
data_groups$code <- as.factor(data_groups$code) #('code' variable refers to participant)
data_groups$sex <- as.factor(data_groups$sex)
glimpse(data_groups) # make sure all counts are numeric
```

```
## Rows: 89
## Columns: 34
## $ code          <fct> 22, 25, 28, 61, 71, 75, 91, 105, 107, 216, 231, 244, 257~
## $ age_ca        <dbl> 8, 9, 9, 7, 5, 10, 9, 5, 12, 11, 10, 10, 12, 12, 6, 6, 1~
## $ age_ca_months <dbl> 107, 110, 112, 93, 68, 121, 117, 70, 150, 139, 129, 127,~
## $ sex           <fct> male, male, male, male, male, male, male, female, male, ~
## $ group         <fct> ASD, ASD, ASD, ASD, ASD, ASD, ASD, ASD, ASD, ASD, ASD, A~
## $ total_ct      <dbl> 303, 201, 200, 256, 213, 220, 233, 256, 271, 276, 275, 2~
## $ mlu           <dbl> 2.910, 3.612, 3.285, 3.922, 2.732, 2.118, 1.880, 2.840, ~
## $ conting       <dbl> 111, 117, 122, 167, 84, 84, 137, 90, 173, 170, 148, 149,~
## $ conting_a     <dbl> 105, 96, 112, 143, 68, 84, 130, 80, 162, 132, 128, 143, ~
## $ conting_q     <dbl> 6, 21, 10, 24, 16, 0, 7, 10, 11, 38, 20, 6, 8, 5, 22, 16~
## $ minimal       <dbl> 119, 57, 54, 63, 37, 82, 58, 65, 72, 67, 86, 54, 121, 14~
## $ minimal_a     <dbl> 48, 13, 14, 27, 8, 11, 17, 20, 39, 20, 24, 25, 56, 78, 3~
## $ minimal_q     <dbl> 71, 44, 40, 36, 29, 71, 41, 45, 33, 47, 62, 29, 65, 71, ~
## $ total_ts      <dbl> 13, 11, 11, 1, 29, 3, 3, 21, 9, 16, 7, 10, 3, 0, 40, 13,~
## $ missing       <dbl> 46, 5, 6, 12, 27, 39, 20, 44, 0, 13, 19, 17, 20, 13, 16,~
## $ other         <dbl> 14, 11, 7, 13, 36, 12, 15, 36, 17, 10, 15, 16, 11, 6, 48~
## $ exp_ass_no    <dbl> 2, 0, 0, 0, 0, 1, 0, 0, 1, 1, 0, 1, 0, 0, 1, 1, 1, 1, 0,~
## $ exp_ass_q     <dbl> 1, 0, 0, 0, 0, 0, 1, 0, 0, 0, 1, 0, 0, 0, 0, 0, 0, 0, 0,~
## $ exp_ass_a     <dbl> 3, 1, 0, 0, 0, 0, 0, 3, 3, 3, 0, 1, 0, 0, 1, 1, 0, 0, 0,~
## $ imp_ass_no    <dbl> 0, 0, 0, 0, 0, 0, 0, 0, 2, 4, 0, 0, 0, 0, 2, 1, 0, 0, 1,~
## $ imp_ass_q     <dbl> 0, 0, 2, 0, 1, 1, 0, 0, 0, 0, 0, 0, 0, 0, 2, 0, 1, 0, 0,~
## $ imp_ass_a     <dbl> 0, 0, 0, 0, 0, 0, 0, 1, 0, 0, 0, 2, 0, 0, 1, 0, 1, 0, 0,~
## $ expl_topre_no <dbl> 1, 0, 0, 0, 0, 0, 0, 0, 0, 0, 2, 0, 0, 0, 3, 1, 0, 0, 0,~
## $ expl_topre_q  <dbl> 0, 0, 1, 0, 0, 0, 1, 1, 0, 0, 0, 0, 0, 0, 3, 1, 0, 1, 1,~
## $ expl_topre_a  <dbl> 2, 0, 2, 0, 0, 0, 0, 4, 3, 3, 1, 0, 1, 0, 5, 2, 1, 0, 0,~
## $ impl_topre_no <dbl> 1, 4, 2, 0, 2, 1, 0, 0, 0, 0, 0, 1, 2, 0, 5, 0, 0, 0, 0,~
## $ impl_topre_q  <dbl> 0, 0, 1, 0, 18, 0, 0, 4, 0, 0, 0, 1, 0, 0, 4, 1, 2, 1, 0~
## $ impl_topre_a  <dbl> 1, 2, 1, 0, 4, 0, 1, 0, 0, 3, 3, 1, 0, 0, 3, 1, 1, 1, 1,~
## $ expl_nass_no  <dbl> 0, 1, 1, 1, 0, 0, 0, 1, 0, 1, 0, 0, 0, 0, 0, 0, 0, 0, 0,~
## $ expl_nass_q   <dbl> 0, 2, 0, 0, 1, 0, 0, 0, 0, 0, 0, 0, 0, 0, 0, 0, 0, 0, 0,~
## $ expl_nass_a   <dbl> 0, 0, 0, 0, 0, 0, 0, 1, 0, 1, 0, 1, 0, 0, 4, 0, 1, 0, 0,~
## $ impl_nass_no  <dbl> 1, 0, 0, 0, 0, 0, 0, 2, 0, 0, 0, 0, 0, 0, 4, 0, 0, 0, 0,~
## $ impl_nass_q   <dbl> 0, 0, 0, 0, 1, 0, 0, 4, 0, 0, 0, 0, 0, 0, 1, 1, 1, 0, 0,~
## $ impl_nass_a   <dbl> 1, 1, 1, 0, 2, 0, 0, 0, 0, 0, 0, 2, 0, 0, 1, 3, 0, 0, 1,~
```

```
# descriptive statistics of participants' characteristics

data_groups %>%
  group_by(group, sex) %>%
  summarise(
   n = n_distinct(code))
```

```
## `summarise()` has grouped output by 'group'. You can override using the
## `.groups` argument.
```

```
## # A tibble: 4 x 3
## # Groups:   group [2]
##   group sex        n
##   <fct> <fct>  <int>
## 1 ASD   female     5
## 2 ASD   male      38
## 3 TD    female    24
## 4 TD    male      22
```

```
data_groups %>%
  group_by(group) %>%
  summarise(word = sum(age_ca), n = n(), 
            Mean = round(mean(age_ca), 2), 
             SD = round(sd(age_ca),2),
             SE = round(SD / sqrt(n), 2))
```

```
## # A tibble: 2 x 6
##   group  word     n  Mean    SD    SE
##   <fct> <dbl> <int> <dbl> <dbl> <dbl>
## 1 ASD     373    43  8.67  2.04  0.31
## 2 TD      388    46  8.43  1.8   0.27
```

Check the groups are well-matched on chronological age
(‘age\_ca\_months’ variable refers to chronological age in months).

```
shapiro.test(data_groups$age_ca_months) #not normally distributed
```

```
## 
##  Shapiro-Wilk normality test
## 
## data:  data_groups$age_ca_months
## W = 0.97019, p-value = 0.03822
```

```
matching_groups <- wilcox.test(age_ca_months ~ group, data = data_groups, exact = FALSE)
print(matching_groups)
```

```
## 
##  Wilcoxon rank sum test with continuity correction
## 
## data:  age_ca_months by group
## W = 1061.5, p-value = 0.5543
## alternative hypothesis: true location shift is not equal to 0
```

# Response types

Extract the descriptive statistics of conversational turns, mlu and
response types (contingent responses, minimal responses, topic shifts,
missing responses and other responses).

```
# conversational turns

data_groups %>%
  group_by(group) %>%
  summarise(word = sum(total_ct), n = n(), 
            Mean = round(mean(total_ct), 2), 
             SD = round(sd(total_ct),2),
             SE = round(SD / sqrt(n), 2))
```

```
## # A tibble: 2 x 6
##   group  word     n  Mean    SD    SE
##   <fct> <dbl> <int> <dbl> <dbl> <dbl>
## 1 ASD   10610    43  247.  42.6   6.5
## 2 TD     8496    46  185.  27.1   4
```

```
# mlu

data_groups %>%
  group_by(group) %>%
  summarise(word = sum(mlu), n = n(), 
            Mean = round(mean(mlu), 2), 
             SD = round(sd(mlu),2),
             SE = round(SD / sqrt(n), 2))
```

```
## # A tibble: 2 x 6
##   group  word     n  Mean    SD    SE
##   <fct> <dbl> <int> <dbl> <dbl> <dbl>
## 1 ASD    149.    43  3.46  0.85  0.13
## 2 TD     154.    46  3.34  0.97  0.14
```

```
# contingent responses

data_groups %>%
  group_by(group) %>%
  summarise(word = sum(conting), n = n(), 
            Mean = round(mean(conting), 2), 
             SD = round(sd(conting),2),
             SE = round(SD / sqrt(n), 2))
```

```
## # A tibble: 2 x 6
##   group  word     n  Mean    SD    SE
##   <fct> <dbl> <int> <dbl> <dbl> <dbl>
## 1 ASD    5219    43 121.   30.9  4.71
## 2 TD     4553    46  99.0  19.9  2.94
```

```
# minimal responses

data_groups %>%
  group_by(group) %>%
  summarise(word = sum(minimal), n = n(), 
            Mean = round(mean(minimal), 2), 
             SD = round(sd(minimal),2),
             SE = round(SD / sqrt(n), 2))
```

```
## # A tibble: 2 x 6
##   group  word     n  Mean    SD    SE
##   <fct> <dbl> <int> <dbl> <dbl> <dbl>
## 1 ASD    3173    43  73.8  28.7  4.38
## 2 TD     3291    46  71.5  22.1  3.26
```

```
# topic shifts 

data_groups %>%
  group_by(group) %>%
  summarise(word = sum(total_ts), n = n(), 
            Mean = round(mean(total_ts), 2), 
             SD = round(sd(total_ts),2),
             SE = round(SD / sqrt(n), 2))
```

```
## # A tibble: 2 x 6
##   group  word     n  Mean    SD    SE
##   <fct> <dbl> <int> <dbl> <dbl> <dbl>
## 1 ASD     483    43 11.2   9.64  1.47
## 2 TD      112    46  2.43  3.62  0.53
```

```
# missing responses

data_groups %>%
  group_by(group) %>%
  summarise(word = sum(missing), n = n(), 
            Mean = round(mean(missing), 2), 
             SD = round(sd(missing),2),
             SE = round(SD / sqrt(n), 2))
```

```
## # A tibble: 2 x 6
##   group  word     n  Mean    SD    SE
##   <fct> <dbl> <int> <dbl> <dbl> <dbl>
## 1 ASD     901    43 21.0  14.1   2.14
## 2 TD      294    46  6.39  6.57  0.97
```

```
# other responses

data_groups %>%
  group_by(group) %>%
  summarise(word = sum(other), n = n(), 
            Mean = round(mean(other), 2), 
             SD = round(sd(other),2),
             SE = round(SD / sqrt(n), 2))
```

```
## # A tibble: 2 x 6
##   group  word     n  Mean    SD    SE
##   <fct> <dbl> <int> <dbl> <dbl> <dbl>
## 1 ASD     834    43 19.4  13.7   2.08
## 2 TD      246    46  5.35  4.48  0.66
```

Perform statistical analyses.

To model children’s MLU, fit a linear regression using the lm
function, with mlu as the dependent variable, and group as fixed effect.
Age (in months) and number of conversational turns were controlled for
by including them as fixed effects.

```
model_mlu <- lm(mlu ~ group + age_ca_months + total_ct, data = data_groups)
summary(model_mlu)
```

```
## 
## Call:
## lm(formula = mlu ~ group + age_ca_months + total_ct, data = data_groups)
## 
## Residuals:
##     Min      1Q  Median      3Q     Max 
## -1.7761 -0.5786 -0.1354  0.6750  2.7000 
## 
## Coefficients:
##                Estimate Std. Error t value Pr(>|t|)    
## (Intercept)    3.660969   0.791372   4.626 1.32e-05 ***
## groupTD       -0.249570   0.260253  -0.959    0.340    
## age_ca_months  0.003419   0.004353   0.785    0.434    
## total_ct      -0.002349   0.002807  -0.837    0.405    
## ---
## Signif. codes:  0 '***' 0.001 '**' 0.01 '*' 0.05 '.' 0.1 ' ' 1
## 
## Residual standard error: 0.9175 on 85 degrees of freedom
## Multiple R-squared:  0.0171, Adjusted R-squared:  -0.01759 
## F-statistic: 0.493 on 3 and 85 DF,  p-value: 0.6881
```

To model counts of response types, fit a mixed-effects negative
binomial regression using the glmer.nb function, with counts as the
dependent variable, and group, response type and their interaction as
fixed effects. By-participant intercepts were included as a random
effect; additionally, age (in months) and number of conversational turns
were controlled for by including them as fixed effects.

```
# transform the dataset into a long format 

glimpse(data_groups)
```

```
## Rows: 89
## Columns: 34
## $ code          <fct> 22, 25, 28, 61, 71, 75, 91, 105, 107, 216, 231, 244, 257~
## $ age_ca        <dbl> 8, 9, 9, 7, 5, 10, 9, 5, 12, 11, 10, 10, 12, 12, 6, 6, 1~
## $ age_ca_months <dbl> 107, 110, 112, 93, 68, 121, 117, 70, 150, 139, 129, 127,~
## $ sex           <fct> male, male, male, male, male, male, male, female, male, ~
## $ group         <fct> ASD, ASD, ASD, ASD, ASD, ASD, ASD, ASD, ASD, ASD, ASD, A~
## $ total_ct      <dbl> 303, 201, 200, 256, 213, 220, 233, 256, 271, 276, 275, 2~
## $ mlu           <dbl> 2.910, 3.612, 3.285, 3.922, 2.732, 2.118, 1.880, 2.840, ~
## $ conting       <dbl> 111, 117, 122, 167, 84, 84, 137, 90, 173, 170, 148, 149,~
## $ conting_a     <dbl> 105, 96, 112, 143, 68, 84, 130, 80, 162, 132, 128, 143, ~
## $ conting_q     <dbl> 6, 21, 10, 24, 16, 0, 7, 10, 11, 38, 20, 6, 8, 5, 22, 16~
## $ minimal       <dbl> 119, 57, 54, 63, 37, 82, 58, 65, 72, 67, 86, 54, 121, 14~
## $ minimal_a     <dbl> 48, 13, 14, 27, 8, 11, 17, 20, 39, 20, 24, 25, 56, 78, 3~
## $ minimal_q     <dbl> 71, 44, 40, 36, 29, 71, 41, 45, 33, 47, 62, 29, 65, 71, ~
## $ total_ts      <dbl> 13, 11, 11, 1, 29, 3, 3, 21, 9, 16, 7, 10, 3, 0, 40, 13,~
## $ missing       <dbl> 46, 5, 6, 12, 27, 39, 20, 44, 0, 13, 19, 17, 20, 13, 16,~
## $ other         <dbl> 14, 11, 7, 13, 36, 12, 15, 36, 17, 10, 15, 16, 11, 6, 48~
## $ exp_ass_no    <dbl> 2, 0, 0, 0, 0, 1, 0, 0, 1, 1, 0, 1, 0, 0, 1, 1, 1, 1, 0,~
## $ exp_ass_q     <dbl> 1, 0, 0, 0, 0, 0, 1, 0, 0, 0, 1, 0, 0, 0, 0, 0, 0, 0, 0,~
## $ exp_ass_a     <dbl> 3, 1, 0, 0, 0, 0, 0, 3, 3, 3, 0, 1, 0, 0, 1, 1, 0, 0, 0,~
## $ imp_ass_no    <dbl> 0, 0, 0, 0, 0, 0, 0, 0, 2, 4, 0, 0, 0, 0, 2, 1, 0, 0, 1,~
## $ imp_ass_q     <dbl> 0, 0, 2, 0, 1, 1, 0, 0, 0, 0, 0, 0, 0, 0, 2, 0, 1, 0, 0,~
## $ imp_ass_a     <dbl> 0, 0, 0, 0, 0, 0, 0, 1, 0, 0, 0, 2, 0, 0, 1, 0, 1, 0, 0,~
## $ expl_topre_no <dbl> 1, 0, 0, 0, 0, 0, 0, 0, 0, 0, 2, 0, 0, 0, 3, 1, 0, 0, 0,~
## $ expl_topre_q  <dbl> 0, 0, 1, 0, 0, 0, 1, 1, 0, 0, 0, 0, 0, 0, 3, 1, 0, 1, 1,~
## $ expl_topre_a  <dbl> 2, 0, 2, 0, 0, 0, 0, 4, 3, 3, 1, 0, 1, 0, 5, 2, 1, 0, 0,~
## $ impl_topre_no <dbl> 1, 4, 2, 0, 2, 1, 0, 0, 0, 0, 0, 1, 2, 0, 5, 0, 0, 0, 0,~
## $ impl_topre_q  <dbl> 0, 0, 1, 0, 18, 0, 0, 4, 0, 0, 0, 1, 0, 0, 4, 1, 2, 1, 0~
## $ impl_topre_a  <dbl> 1, 2, 1, 0, 4, 0, 1, 0, 0, 3, 3, 1, 0, 0, 3, 1, 1, 1, 1,~
## $ expl_nass_no  <dbl> 0, 1, 1, 1, 0, 0, 0, 1, 0, 1, 0, 0, 0, 0, 0, 0, 0, 0, 0,~
## $ expl_nass_q   <dbl> 0, 2, 0, 0, 1, 0, 0, 0, 0, 0, 0, 0, 0, 0, 0, 0, 0, 0, 0,~
## $ expl_nass_a   <dbl> 0, 0, 0, 0, 0, 0, 0, 1, 0, 1, 0, 1, 0, 0, 4, 0, 1, 0, 0,~
## $ impl_nass_no  <dbl> 1, 0, 0, 0, 0, 0, 0, 2, 0, 0, 0, 0, 0, 0, 4, 0, 0, 0, 0,~
## $ impl_nass_q   <dbl> 0, 0, 0, 0, 1, 0, 0, 4, 0, 0, 0, 0, 0, 0, 1, 1, 1, 0, 0,~
## $ impl_nass_a   <dbl> 1, 1, 1, 0, 2, 0, 0, 0, 0, 0, 0, 2, 0, 0, 1, 3, 0, 0, 1,~
```

```
resp_types <- gather(data_groups, key = "type", value="count", 8,11,14,15,16)
resp_types$type <- as.factor(resp_types$type)
View(resp_types)

# run the model

model_responses <- glmer.nb(count ~ group * type + age_ca_months + total_ct + (1 | code), data = resp_types)
```

```
## boundary (singular) fit: see help('isSingular')
```

```
summary(model_responses)
```

```
## Generalized linear mixed model fit by maximum likelihood (Laplace
##   Approximation) [glmerMod]
##  Family: Negative Binomial(3.9665)  ( log )
## Formula: count ~ group * type + age_ca_months + total_ct + (1 | code)
##    Data: resp_types
## 
##      AIC      BIC   logLik deviance df.resid 
##   3475.2   3532.6  -1723.6   3447.2      431 
## 
## Scaled residuals: 
##     Min      1Q  Median      3Q     Max 
## -1.8055 -0.7608 -0.1932  0.4712  5.0365 
## 
## Random effects:
##  Groups Name        Variance Std.Dev. 
##  code   (Intercept) 6.43e-12 2.536e-06
## Number of obs: 445, groups:  code, 89
## 
## Fixed effects:
##                        Estimate Std. Error z value Pr(>|z|)    
## (Intercept)           4.3498023  0.2302601  18.891  < 2e-16 ***
## groupTD               0.0588977  0.1177990   0.500    0.617    
## typeminimal          -0.4982282  0.1107345  -4.499 6.82e-06 ***
## typemissing          -1.7645590  0.1144954 -15.412  < 2e-16 ***
## typeother            -1.8847203  0.1151091 -16.373  < 2e-16 ***
## typetotal_ts         -2.4489582  0.1192714 -20.533  < 2e-16 ***
## age_ca_months        -0.0060026  0.0011987  -5.008 5.51e-07 ***
## total_ct              0.0045084  0.0007732   5.831 5.52e-09 ***
## groupTD:typeminimal   0.1698226  0.1541194   1.102    0.271    
## groupTD:typemissing  -0.9808326  0.1665609  -5.889 3.89e-09 ***
## groupTD:typeother    -1.0640926  0.1688425  -6.302 2.93e-10 ***
## groupTD:typetotal_ts -1.3135336  0.1852848  -7.089 1.35e-12 ***
## ---
## Signif. codes:  0 '***' 0.001 '**' 0.01 '*' 0.05 '.' 0.1 ' ' 1
## 
## Correlation of Fixed Effects:
##             (Intr) gropTD typmnm typmss typthr typtt_ ag_c_m ttl_ct grpTD:typmn
## groupTD     -0.530                                                             
## typeminimal -0.219  0.460                                                      
## typemissing -0.265  0.455  0.477                                               
## typeother   -0.238  0.435  0.475  0.463                                        
## typetotl_ts -0.230  0.415  0.458  0.447  0.449                                 
## age_c_mnths -0.468 -0.025 -0.015  0.044  0.068  0.087                          
## total_ct    -0.737  0.388 -0.012  0.011 -0.038 -0.050 -0.151                   
## grpTD:typmn  0.168 -0.648 -0.718 -0.343 -0.341 -0.329  0.006 -0.001            
## grpTD:typms  0.124 -0.590 -0.330 -0.684 -0.315 -0.304  0.026  0.023  0.459     
## grpTD:typth  0.135 -0.582 -0.325 -0.313 -0.678 -0.302  0.003  0.023  0.452     
## grpTD:typt_  0.136 -0.537 -0.296 -0.286 -0.284 -0.638  0.002  0.006  0.412     
##             grpTD:typms grpTD:typt
## groupTD                           
## typeminimal                       
## typemissing                       
## typeother                         
## typetotl_ts                       
## age_c_mnths                       
## total_ct                          
## grpTD:typmn                       
## grpTD:typms                       
## grpTD:typth  0.419                
## grpTD:typt_  0.382       0.376    
## optimizer (Nelder_Mead) convergence code: 0 (OK)
## boundary (singular) fit: see help('isSingular')
```

```
# run the model including sex

model_responses_2 <- glmer.nb(count ~ group * type + age_ca_months + total_ct + sex + (1 | code), data = resp_types)
```

```
## boundary (singular) fit: see help('isSingular')
```

```
summary(model_responses_2)
```

```
## Generalized linear mixed model fit by maximum likelihood (Laplace
##   Approximation) [glmerMod]
##  Family: Negative Binomial(3.9802)  ( log )
## Formula: count ~ group * type + age_ca_months + total_ct + sex + (1 |  
##     code)
##    Data: resp_types
## 
##      AIC      BIC   logLik deviance df.resid 
##   3474.6   3536.0  -1722.3   3444.6      430 
## 
## Scaled residuals: 
##     Min      1Q  Median      3Q     Max 
## -1.8083 -0.7602 -0.1917  0.4493  5.2569 
## 
## Random effects:
##  Groups Name        Variance  Std.Dev. 
##  code   (Intercept) 7.729e-11 8.791e-06
## Number of obs: 445, groups:  code, 89
## 
## Fixed effects:
##                        Estimate Std. Error z value Pr(>|z|)    
## (Intercept)           4.3149771  0.2308088  18.695  < 2e-16 ***
## groupTD               0.0921012  0.1193556   0.772    0.440    
## typeminimal          -0.4974838  0.1105610  -4.500 6.81e-06 ***
## typemissing          -1.7656332  0.1143338 -15.443  < 2e-16 ***
## typeother            -1.8879897  0.1149683 -16.422  < 2e-16 ***
## typetotal_ts         -2.4515293  0.1191293 -20.579  < 2e-16 ***
## age_ca_months        -0.0061700  0.0012019  -5.133 2.85e-07 ***
## total_ct              0.0043407  0.0007783   5.577 2.44e-08 ***
## sexmale               0.1082026  0.0664545   1.628    0.103    
## groupTD:typeminimal   0.1696560  0.1538750   1.103    0.270    
## groupTD:typemissing  -0.9829002  0.1663600  -5.908 3.46e-09 ***
## groupTD:typeother    -1.0682439  0.1686665  -6.333 2.40e-10 ***
## groupTD:typetotal_ts -1.3153358  0.1851107  -7.106 1.20e-12 ***
## ---
## Signif. codes:  0 '***' 0.001 '**' 0.01 '*' 0.05 '.' 0.1 ' ' 1
```

```
## 
## Correlation matrix not shown by default, as p = 13 > 12.
## Use print(x, correlation=TRUE)  or
##     vcov(x)        if you need it
```

```
## optimizer (Nelder_Mead) convergence code: 0 (OK)
## boundary (singular) fit: see help('isSingular')
```

```
anova(model_responses, model_responses_2) # model_responses is the optimal model
```

```
## Data: resp_types
## Models:
## model_responses: count ~ group * type + age_ca_months + total_ct + (1 | code)
## model_responses_2: count ~ group * type + age_ca_months + total_ct + sex + (1 | code)
##                   npar    AIC    BIC  logLik deviance  Chisq Df Pr(>Chisq)
## model_responses     14 3475.2 3532.6 -1723.6   3447.2                     
## model_responses_2   15 3474.6 3536.0 -1722.3   3444.6 2.6382  1     0.1043
```

To assess which conditions differed significantly, perform post-hoc
(emmeans) analyses.

```
emmeans(model_responses, pairwise~list(group|type, type|group), adjust="Tukey")
```

```
## $emmeans
## type = conting:
##  group emmean     SE  df asymp.LCL asymp.UCL
##  ASD    4.666 0.0815 Inf     4.507      4.83
##  TD     4.725 0.0788 Inf     4.571      4.88
## 
## type = minimal:
##  group emmean     SE  df asymp.LCL asymp.UCL
##  ASD    4.168 0.0829 Inf     4.006      4.33
##  TD     4.397 0.0790 Inf     4.242      4.55
## 
## type = missing:
##  group emmean     SE  df asymp.LCL asymp.UCL
##  ASD    2.902 0.0866 Inf     2.732      3.07
##  TD     1.980 0.0987 Inf     1.786      2.17
## 
## type = other:
##  group emmean     SE  df asymp.LCL asymp.UCL
##  ASD    2.782 0.0888 Inf     2.608      2.96
##  TD     1.776 0.1010 Inf     1.579      1.97
## 
## type = total_ts:
##  group emmean     SE  df asymp.LCL asymp.UCL
##  ASD    2.217 0.0944 Inf     2.033      2.40
##  TD     0.963 0.1220 Inf     0.724      1.20
## 
## Results are given on the log (not the response) scale. 
## Confidence level used: 0.95 
## 
## $contrasts
## type = conting:
##  contrast estimate    SE  df z.ratio p.value
##  ASD - TD  -0.0589 0.118 Inf  -0.500  0.6171
## 
## type = minimal:
##  contrast estimate    SE  df z.ratio p.value
##  ASD - TD  -0.2287 0.119 Inf  -1.927  0.0540
## 
## type = missing:
##  contrast estimate    SE  df z.ratio p.value
##  ASD - TD   0.9219 0.136 Inf   6.783  <.0001
## 
## type = other:
##  contrast estimate    SE  df z.ratio p.value
##  ASD - TD   1.0052 0.139 Inf   7.252  <.0001
## 
## type = total_ts:
##  contrast estimate    SE  df z.ratio p.value
##  ASD - TD   1.2546 0.157 Inf   7.971  <.0001
## 
## Results are given on the log (not the response) scale.
```

Extract model predictions.

```
pred_responses <- ggeffects::ggeffect(model_responses, terms = c("group", "type"))
```

```
## You are calculating adjusted predictions on the population-level (i.e.
##   `type = "fixed"`) for a *generalized* linear mixed model.
##   This may produce biased estimates due to Jensen's inequality. Consider
##   setting `bias_correction = TRUE` to correct for this bias.
##   See also the documentation of the `bias_correction` argument.
```

```
pred_responses <- tibble::as_tibble(pred_responses)
pred_responses <- pred_responses %>%
  rename(group = x, Type = group)

pred_responses
```

```
## # A tibble: 10 x 6
##    group predicted std.error conf.low conf.high Type    
##    <fct>     <dbl>     <dbl>    <dbl>     <dbl> <fct>   
##  1 ASD      106.      0.0815    90.6     125.   conting 
##  2 TD       113.      0.0788    96.6     132.   conting 
##  3 ASD       64.6     0.0829    54.9      76.0  minimal 
##  4 TD        81.2     0.0790    69.5      94.8  minimal 
##  5 ASD       18.2     0.0866    15.4      21.6  missing 
##  6 TD         7.24    0.0987     5.97      8.79 missing 
##  7 ASD       16.1     0.0888    13.6      19.2  other   
##  8 TD         5.91    0.101      4.85      7.20 other   
##  9 ASD        9.18    0.0944     7.63     11.1  total_ts
## 10 TD         2.62    0.122      2.06      3.33 total_ts
```

Plot the total counts of response types per participant and group
with fitted values and 95% confidence intervals.

```
resp_types <- resp_types %>% 
  mutate(type_dodge = case_when(
    group == "TD" ~ as.numeric(type) + 0.22,
    group == "ASD" ~ as.numeric(type) - 0.22
  ))

ggplot() +
  geom_jitter(data = resp_types, aes(x = type_dodge, y = count, color = group), 
               height = 0, width = 0.1, size = 1.5, alpha = 0.3, show.legend = TRUE) +
   geom_point(data = pred_responses, 
             aes(x = Type, y = predicted, color = group), 
             size = 4, shape = 20, fill = "white", stroke = 1, position = position_dodge(width = 0.9), show.legend = TRUE) +
  geom_errorbar(data = pred_responses, 
                aes(x = Type, ymin = conf.low, ymax = conf.high, group = group, color = group),  
                width = 0.5, linetype = 1,  
                position = position_dodge(width = 0.9), size = 1.2, show.legend = FALSE) +
  labs(x = "", y = "Total counts", title = "",  caption = "Figure 1. Total counts of response types per participant and group with fitted values and 95% confidence intervals.") +
  theme_minimal() +
  scale_fill_manual(values = c("ASD" = "mediumorchid3", "TD" = "darkolivegreen3"), 
                    labels = c("ASD" = "Autistic", "TD" = "Neurotypical"), name = NULL) +
  scale_color_manual(values = c("ASD" = "mediumorchid3", "TD" = "darkolivegreen3"), 
                     labels = c("ASD" = "Autistic", "TD" = "Neurotypical"), name = NULL) +
  theme(axis.text.x = element_text(angle = 360, hjust = 1.5), legend.position = "top",
        plot.caption = element_text(size = 20, hjust = 0.5, face = "italic")) + 
  scale_y_continuous(limits = c(0, 160), expand = c(0, 0.0)) + 
  theme_bw() + 
  scale_x_discrete(labels = c("conting" = "Contingent", "minimal" = "Minimal", 
                               "total_ts" = "Topic shift", "missing" = "Missing", 
                               "other" = "Other")) + 
  annotate("text", x = 3, y = 45, label = "***", size = 4, color = "black") +  
  annotate("text", x = 4, y = 65, label = "***", size = 4, color = "black") +  
  annotate("text", x = 5, y = 54, label = "***", size = 4, color = "black") +  
  geom_segment(aes(x = 3.75, xend = 4.25, y = 63, yend = 63), color = "black", size = 0.5) + 
  geom_segment(aes(x = 2.75, xend = 3.25, y = 43, yend = 43), color = "black", size = 0.5) + 
  geom_segment(aes(x = 4.75, xend = 5.25, y = 52, yend = 52), color = "black", size = 0.5)
```

```
## Warning: Using `size` aesthetic for lines was deprecated in ggplot2 3.4.0.
## i Please use `linewidth` instead.
## This warning is displayed once every 8 hours.
## Call `lifecycle::last_lifecycle_warnings()` to see where this warning was
## generated.
```

```
## Warning: No shared levels found between `names(values)` of the manual scale and the
## data's fill values.
```

```
## Warning: Removed 6 rows containing missing values or values outside the scale range
## (`geom_point()`).
```

# Topic shift types

Create a new dataset in which a score (1, 2 or 3) is assigned to each
topic shift based on the neurotypical adults’ naturalness judgments and
the cluster analysis performed (see Supplementary Material 2).

```
# transform the previous dataset

data_ts_types <- gather(data_groups, key = "ts_type", value="ts_count", 17:34)

data_ts_types$ts_type <- as.factor(data_ts_types$ts_type)

View(data_ts_types)

data_ts_types <- data_ts_types[rep(1:nrow(data_ts_types), times = data_ts_types$ts_count), ]

View(data_ts_types)

# assign a score to each topic shift type

data_ts_types <- data_ts_types %>% 
  mutate(
    score = case_when(
      ts_type %in% c("expl_nass_a", "impl_nass_a", "expl_nass_q", "impl_nass_q", "impl_nass_no") ~ 1,
      ts_type %in% c("exp_ass_q", "imp_ass_q", "exp_ass_a", "expl_topre_q", "impl_topre_q", 
                     "expl_topre_a", "impl_topre_a", "impl_topre_no", "expl_nass_no") ~ 2,
      ts_type %in% c("exp_ass_no", "imp_ass_no", "imp_ass_a", "expl_topre_no") ~ 3,    )
  )

View(data_ts_types)
```

Extract the total counts of topic shift types per group.

```
data_ts_types %>%
  group_by(group, score) %>%
  summarise(
    count = n())
```

```
## `summarise()` has grouped output by 'group'. You can override using the
## `.groups` argument.
```

```
## # A tibble: 6 x 3
## # Groups:   group [2]
##   group score count
##   <fct> <dbl> <int>
## 1 ASD       1    99
## 2 ASD       2   285
## 3 ASD       3    99
## 4 TD        1    20
## 5 TD        2    50
## 6 TD        3    42
```

Perform statistical analyses. Perform a linear mixed-effects
regression using the lmer function, with scores as dependent variable,
group as fixed effect, and participant as a random effect. Age (in
months) and number of conversational turns are included as control
variables.

```
model_ts_types <- lmer(score ~ group + age_ca_months + total_ct + (1|code), data = data_ts_types)

summary(model_ts_types)
```

```
## Linear mixed model fit by REML. t-tests use Satterthwaite's method [
## lmerModLmerTest]
## Formula: score ~ group + age_ca_months + total_ct + (1 | code)
##    Data: data_ts_types
## 
## REML criterion at convergence: 1198.3
## 
## Scaled residuals: 
##      Min       1Q   Median       3Q      Max 
## -1.98246 -0.34865 -0.00327  0.42751  2.01046 
## 
## Random effects:
##  Groups   Name        Variance Std.Dev.
##  code     (Intercept) 0.0285   0.1688  
##  Residual             0.3991   0.6317  
## Number of obs: 595, groups:  code, 68
## 
## Fixed effects:
##                Estimate Std. Error        df t value Pr(>|t|)    
## (Intercept)   1.560e+00  2.721e-01 4.565e+01   5.735 7.39e-07 ***
## groupTD       2.420e-01  9.897e-02 6.857e+01   2.445   0.0171 *  
## age_ca_months 3.539e-03  1.532e-03 4.698e+01   2.311   0.0253 *  
## total_ct      3.724e-04  8.549e-04 3.671e+01   0.436   0.6657    
## ---
## Signif. codes:  0 '***' 0.001 '**' 0.01 '*' 0.05 '.' 0.1 ' ' 1
## 
## Correlation of Fixed Effects:
##             (Intr) gropTD ag_c_m
## groupTD     -0.545              
## age_c_mnths -0.580  0.102       
## total_ct    -0.794  0.523 -0.010
```

Extract model predictions.

```
pred_ts <- ggeffects::ggeffect(model_ts_types, terms = "group")
pred_ts <- tibble::tibble(pred_ts)
pred_ts <- pred_ts %>% dplyr::mutate(group = x)

pred_ts
```

```
## # A tibble: 2 x 6
##   x     predicted std.error conf.low conf.high group
##   <fct>     <dbl>     <dbl>    <dbl>     <dbl> <fct>
## 1 ASD        2.01    0.0430     1.92      2.09 ASD  
## 2 TD         2.25    0.0863     2.08      2.42 TD
```

Plot the of naturalness scores per group with fitted values and 95%
confidence intervals.

```
ggplot2::ggplot() + 
 ggplot2::geom_violin(data = data_ts_types, 
                       ggplot2::aes(x = group, y = score, color = group), 
                       show.legend = FALSE, alpha = 0.4, trim = TRUE, size = 1) + 
  ggplot2::geom_point(data = pred_ts, 
                      ggplot2::aes(x = group, y = predicted, color = group, fill = group), 
                      size = 5, shape = 20, stroke = 2, 
                      position = ggplot2::position_dodge(width = 0.45), show.legend = FALSE) +
  ggplot2::geom_errorbar(data = pred_ts, 
                         ggplot2::aes(x = group, ymin = conf.low, ymax = conf.high, color = group), 
                         size = 1, width = 0.10, linetype = 1, 
                         show.legend = FALSE, position = ggplot2::position_dodge(width = 0.45)) + 
  ggplot2::theme_bw() + 
  ggplot2::theme(plot.caption = ggplot2::element_text(face = "italic"), 
                 axis.line.y.right = ggplot2::element_line(size = 0.75, 
                                                          arrow = grid::arrow(length = ggplot2::unit(0.3, "cm"), ends = "both")), 
                 axis.title.y.right = ggplot2::element_text(angle = 0), 
                 axis.text.y.right = ggplot2::element_blank(), 
                 axis.ticks.y.right = ggplot2::element_blank()) + 

  ggplot2::scale_color_manual(values = c("ASD" = "mediumorchid3", "TD" = "darkolivegreen3"), 
                              labels = c("ASD" = "Autistic", "TD" = "Neurotypical"), name = NULL) +
  ggplot2::scale_fill_manual(values = c("ASD" = "mediumorchid3", "TD" = "darkolivegreen3"), 
                             labels = c("ASD" = "Autistic", "TD" = "Neurotypical"), name = NULL) +

  ggplot2::labs(title = "", x = "", y = "Score", caption = "Figure 2. Distribution of topic shift naturalness scores per group with fitted values and 95% confidence intervals.") + 

  ggplot2::coord_cartesian(ylim = c(1, 3.2)) +

  ggplot2::scale_y_continuous(breaks = seq(1, 3, by = 1)) + 
 ggplot2::scale_x_discrete(labels = c("ASD" = "Autistic", "TD" = "Neurotypical")) +
 ggplot2::theme(plot.caption = ggplot2::element_text(face = "plain", size = 10, hjust = 0.5),  
                 axis.line.y.right = ggplot2::element_line(size = 0.75, 
                                                          arrow = grid::arrow(length = ggplot2::unit(0.3, "cm"), ends = "both")), 
                 axis.title.y.right = ggplot2::element_text(angle = 0), 
                 axis.text.y.right = ggplot2::element_blank(), 
                 axis.ticks.y.right = ggplot2::element_blank())
```

```
## Warning: The `size` argument of `element_line()` is deprecated as of ggplot2 3.4.0.
## i Please use the `linewidth` argument instead.
## This warning is displayed once every 8 hours.
## Call `lifecycle::last_lifecycle_warnings()` to see where this warning was
## generated.
```
