# Supplementary material for: Conversational Topic Shifts and Topic Maintenance in Autistic and Neurotypical Children
Source: Autism Res. 2026 Feb 18;19(4):e70204. doi: 10.1002/aur.70204 (PMC13087834; doi:10.1002/aur.70204)
Supplement: Supplementary file 1 — Data S1: Supporting Information. [file AUR-19-0-s001.zip › Supplementary Materials/Supplementary Material 1_Group comparisons/Supplementary Material 1_Coding.docx]

**Supplementary Material 1. Coding protocol**

**Transcription**

Each production was manually transcribed following the CHAT transcription format, the standard transcription system for the TalkBank and Child Language Data Exchange System (CHILDES) projects (MacWhinney, 2000). The CHAT manual can be retrieved from the Talkbank webpage: <https://talkbank.org/>. All transcriptions and coding were done using the *Computerized Language ANalysis* (CLAN) program, which can also be downloaded from the Talkbank webpage: <https://talkbank.org/>.

**Coding scheme**

We coded children’s responses to the experimenter’s preceding conversational turn. Each coding category (i.e., response type) is described in detail and illustrated with examples below (in the examples, ‘E’ stands for Experimenter, and ‘C’ stands for Child). The examples are drawn from actual exchanges between the experimenter and the child during the administration of the *Eliciting Language Samples for Analysis* (ELSA) elicitation protocol, developed by Dr. Helen Tager-Flusberg and colleagues at Boston University in 2016 (the protocol is available on <https://sites.bu.edu/elsa/elsa-2/>). The conversations originally took place in Spanish but have been translated into English.

**1. Topic shifts**

We developed a novel protocol to code topic shifts in conversation. Responses that deviate from the topic of discussion of the immediately preceding utterance are coded under this category.^[[1]](#footnote-1)^ A topic shift can take the form of a declarative, interrogative, exclamative or imperative clause. Drawing on the *Question Under Discussion* discourse theory (Roberts, 2012; Onea, 2013, 2016; Riester, 2019) and Van Kuppevelt (1995)’s work, we distinguished three features of each topic shift: *association*, *marking* and *prompt*. The goal of this coding scheme is to provide a fine-grained characterization of the different topic shifts that can occur in conversation. For a more theoretical and technical discussion of these categories, see AUTHORS (forthcoming).

**1.1. Association**

This category captures the thematic relationship between a newly introduced topic and prior topics, and is largely based on the distinction proposed by Van Kuppevelt (1995).

**1.1.1. Associated**

Topic shifts are coded as associated if they are thematically related to the immediate previous conversation topic. For example:

1. E: Oh, do you live in San Miguel?

C: Yes.

E: I knew it.

C: And you?

E: I live here, near the town hall.

C: Ah. **Do you know how my mom refers to town halls?**

- - 1. **Topic reintroduction**

A topic reintroduction is a topic shift which is related to a previously discussed topic in the conversation. However, unlike in associated topic shifts, this topic is not present in the immediately previous utterance and has already been closed. Identifying the association thus requires revisiting earlier parts of the conversation. For example:

1. E: I live in Basauri, near Bilbao. Have you ever been to Bilbao?

C: Yes, a few times. I’ve been to the beach, to the rocket…

E: The rocket?

C: Yes.

E: What rocket?

C: **Why is it called Basauri?**

- - 1. **Non-associated**

Topic shifts unrelated to any previous topic raised in the conversation are coded as non-associated. For example:

1. E: Do you know where your cap is from?

C: From Catalonia?

E: No. It’s from Sopelana. Do you know where Sopelana is?

C: **My teacher always lets me use the computer when I’ve done my work.**

- 1. **Marking**

This category captures the presence/absence of a discourse marker that signals the topic shift. Example (4) below is a case of an explicitly marked topic shift, while (3) above, repeated in (5) below, illustrates an implicit topic shift.

**1.2.1. Explicit**

(4) E: Who did you go hiking with?

C: With many teachers.

E: Ah, with your school teachers? I thought you had gone with your family.

C: Yes. **Hey, look, I have a video game called ‘spy camera’.**

- - 1. **Implicit**

(5) E: Do you know where your cap is from?

C: From Catalonia?

E: No. It’s from Sopelana. Do you know where Sopelana is?

C: **My teacher always lets me use the computer when I’ve done my work.**

- 1. **Prompt**

We developed the category *prompt* to account for the context in which the topic shift occurs, specifically accounting for the type of experimenter utterance immediately preceding the shift.

**1.3.1. None**

A topic shift is coded as having no prompt when the child first addresses the interlocutor’s previous topic and then subsequently introduces a new topic. This type of topic shift is illustrated in (4) above, repeated in (6) below:

(6) E: Who did you go hiking with?

C: With many teachers.

E: Ah, with your school teachers? I thought you had gone with your family.

C: Yes. **Hey, look, I have a video game called ‘spy camera’.**

Here, C responds to E’s previous question (i.e., C utters *Yes*) before shifting the conversation in a new direction.

This category also includes cases in which the child shifts topic after an experimenter’s utterance that suggests the closure of the previous topic (i.e., an utterance that does not prompt the child to elaborate on the topic). For example:

(7) E: Were you looking forward to starting school, or would you have preferred to stay on vacation a bit longer?

C: I was looking forward to it to be with my friends.

E: Of course. Didn’t you see them during the summer?

C: No. Well, when summer ended, I saw my friend Aitor.

E: Ah, cool.

C: **Do you know anyone named Aitor?**

In (7), E responds with an utterance that does not invite further elaboration (*Ah, cool*). C produces a topic shift in the following turn.

- - 1. **Question**

The child produces a topic shift immediately after the experimenter’s question, as in examples (2-3) above, repeated in (8-9) below:

(8) E: I live in Basauri, near Bilbao. Have you ever been to Bilbao?

C: Yes, a few times. I’ve been to the beach, to the rocket…

E: The rocket?

C: Yes.

E: What rocket?

C: **Why is it called Basauri?**

(9) E: Do you know where your cap is from?

C: From Catalonia?

E: No. It’s from Sopelana. Do you know where Sopelana is?

C: **My teacher always lets me use the computer when I’ve done my work.**

In these cases, E utters a question that expects an answer, but C leaves the question unanswered and shifts topic.

- - 1. **Statement**

The child produces a topic shift immediately after the experimenter’s statement, as shown in (10) below:

(10) E: Have you ever planted anything?

C: At school, we planted peas and other things.

E: My grandma has a vegetable garden, and she grows peppers and things like that.

C: **Our teacher has a vegetable garden and a mosquito bit her on the arm.**

**Examples of topic shift types**

The combination of the categories in our coding scheme resulted in 18 different types of topic shifts. An example of each is shown below:

*Associated – explicit – none*

E: Oh, do you live in San Miguel?

C: Yes.

E: I knew it.

C: And you?

E: I live here, near the town hall.

C: Ah. **Do you know how my mom refers to town halls?**

*Associated – explicit – question*

E: When you go hiking, what do you carry in your backpack?

C: Actually, I don’t even know what I take…

E: Okay, what would you take with you when you go hiking?

C: A canteen.

E: A canteen to drink water, right? What else?

C: **Speaking of thirst, I’m starting to feel a bit thirsty.**

*Associated – explicit – statement*

E: Hey, what kind of tree do you think it is?

C: I don’t know.

E: This is a walnut tree. Walnut trees bear walnuts. Do you like walnuts?

C: Yes.

E: My grandma has a vegetable garden, and she gives me loads of walnuts every time I visit her.

C: **You know what? I think I found one that’s spoiled.**

*Associated – implicit – none*

E: Were you looking forward to starting school, or would you have preferred to stay on vacation a bit longer?

C: I was looking forward to it to be with my friends.

E: Of course. Didn’t you see them during the summer?

C: No. Well, when summer ended, I saw my friend Aitor.

E: Ah, cool.

C: **Do you know anyone named Aitor?**

*Associated – implicit – question*

E: I think something is wrong with these animals. They look a bit sad.

C: The little donkey has his head down. He’s crying.

E: Is he crying? Why?

C: I don’t know.

E: Do you think his leg hurts?

C: **The horse’s leg is already healed.**

*Associated – implicit – statement*

E: Have you ever planted anything?

C: At school, we planted peas and other things.

E: My grandma has a vegetable garden, and she grows peppers and things like that.

C: **Our teacher has a vegetable garden and a mosquito bit her on the arm**.

*Topic reintroduction – explicit – none*

E: What’s your robin’s name?

C: His name is, uh… I need to think.

E: Have you forgotten? It’s okay. Do you also have siblings or are you an only child?

C: I’m an only child. I have cousins.

E: Alright, so your family is pretty big.

C: Ah, yes. **Ah, I remember what he’s called now. He’s called Ruisi.**

*Topic reintroduction – explicit – question*

E: I have the gray marker. Do you want to borrow it?

C: Yes, yes, yes.

E: Well, you told me you were with Julia too. Did you have fun with her?

C: Yes, very much.

E: Hey, and these days when you don’t have school, what do you do at home?

C: **Wait, just a bit more gray.**

*Topic reintroduction – explicit – statement*

E: What mountain have you been to?

C: Well, it’s close to Landa.

E: Hey, what do you usually take with you when you go hiking?

C: Some food.

E: Ah, a sandwich, you told me earlier.

C: **And I’ve been to a mountain in Barria**.

*Topic reintroduction – implicit – none*

E: What happened to this tree?

C: All its leaves and walnuts have fallen because of autumn.

E: That’s right, leaves fall in autumn. Your birthday is in summer, right?

C: Yes.

E: My birthday is in autumn, near Halloween.

C: I see. **How should I plant the walnuts?**

*Topic reintroduction – implicit – question*

E: I live in Basauri, near Bilbao. Have you ever been to Bilbao?

C: Yes, a few times. I’ve been to the beach, to the rocket…

E: The rocket?

C: Yes.

E: What rocket?

C: **Why is it called Basauri?**

*Topic reintroduction – implicit – statement*

E: What will grow if we plant a walnut?

C: A walnut tree.

E: That’s right. Come on, help me plant them. Great job!

C: Done.

E: Now what we’re going to do is check these animals out.

C: **The walnuts are spoiled.**

*Non-associated – explicit – none*

E: Who did you go hiking with?

C: With many teachers.

E: Ah, with your school teachers? I thought you had gone with your family.

C: Yes. **Hey, look, I have a video game called ‘spy camera’.**

*Non-associated – explicit – question*

E: Look what I have. What’s this?

C: A compass. It shows you the north, south, east and west.

E: Do you know how to use it?

C: Well, no, because I don’t know which direction my house is in.

E: Of course. Alright, let’s go. Do you want to carry the backpack?

C: **Ah, one thing. Do you know one of the things that scares me the most? Heights.**

*Non-associated – explicit – statement*

E: With the balls it’s harder to hit the target. Do you want to try with a dart?

C: Yes.

E: Come a bit closer.

C: Well, look, I hit the number ten!

E: Ten! Then I’ve won.

C: **Look, a zebra.**

*Non-associated – implicit – none*

E: Well, I like spring more than summer.

C: I don’t.

E: What do you usually do in the summer? Do you usually go on vacation?

C: Mhm.

E: Yes?

C: Yes. **I’m going to open the fridge.**

*Non-associated – implicit – question*

E: Do you know where your cap is from?

C: From Catalonia?

E: No. It’s from Sopelana. Do you know where Sopelana is?

C: **My teacher always lets me use the computer when I’ve done my work.**

*Non-associated – implicit – statement*

E: Okay, it’s autumn we’ve said, right?

C: Yes.

E: Your birthday is in the summer, right?

C: Yes.

E: My birthday is in autumn.

C: **What do I do with this?**

**2. Responses that do not involve a topic shift**

Building on Abbot-Smith et al. (2021)’s coding protocol (available on <https://osf.io/q7wa4/>), we categorized the remaining responses (i.e., those in which the child did not shift topic) into the following types: *contingent, minimal, missing* and *other*.

**2.1. Contingent responses**

Contingent responses are (declarative, interrogative, exclamative or imperative) responses that maintain and elaborate on the previous topic. As described by Abbot-Smith et al. (2021:3), these “provide an appropriate, informative, on-topic response which adds information to the experimenter’s previous utterance, thus providing the experimenter with an opportunity to follow in on the child’s utterance”. Children’s responses to both the experimenter’s previous statement (see (11) below) and question (see (12) below) are coded under this category.

(11) E: We’re going to draw something here.

C: **And what can we draw?**

(12) E: Do you spend time with your family on holidays?

C: **Yes, and with my friends as well.**

**2.2. Minimal responses**

“Minimal responses are those which are not off-topic, but which do not add information to the preceding conversation turn and thus they do not provide the conversation partner with an opportunity to follow in” (Abbot-Smith et al., 2021:5-7). The following responses are coded under this category, which is primarily based on Abbot-Smith and colleagues’ protocol (though with some modifications, such as the inclusion of non-verbal responses): (i) Short utterances with semantic content (e.g., E: *I have a dog*. C: *Oh, a doggy*); (ii) short utterances empty of semantic content (e.g., *Ah*); (iii) non-word noises (e.g., *Mmm*); (iv) phrases which provide an affective comment on what the experimenter said but do not provide the experimenter with an opportunity to then follow in on them (e.g., *Did you?*); (v) repetitions of what the experimenter said (e.g., E: *It’s a dog*. C: *A dog*); (vi); *yes/no* responses to polar questions (E: *Do you like chocolate?* C: *Yes*); (vii) non-verbal responses (e.g., nodding, laughing); (viii) incomplete utterances (e.g., *So…*).

**2.3. Missing responses**

*Missing responses* are null responses to the experimenter’s immediately preceding turn. Following Pagmar et al. (2022), a missing response is coded when (i) >2 seconds have passed after the experimenter’s turn, (ii) the child is not offering any (non-)verbal response, and (iii) the experimenter once again takes a turn. Note that, in our protocol, non-verbal responses which can clearly be interpreted in relation to the preceding turn are coded under the *minimal responses* category.

**2.4. Other responses**

Responses not fitting into any other category are coded as *other responses* (Abbot-Smith et al., 2021). These include, for instance, (i) utterances that are unintelligible to the point of preventing coding; (ii) utterances that can potentially be coded in multiple categories; (iii) clarification questions from the child (e.g., *What does that mean?*); (iv) underinformative answers to questions, in which the child provides less information than is needed or expected in a given context (e.g., E: *What can we do apart from picking up the leaves?* C: *Pick them up*).

**Coding software**

As mentioned at the beginning of this document, coding was done using the CLAN program. The CLAN manual was followed for the creation of our own variables (the manual can be downloaded from the Talkbank webpage: <https://talkbank.org/>). After coding the transcripts, the variable counts were extracted using the ‘freq’ command.

**References**

Abbot-Smith, K., Matthews, D., Malkin, L., & Nice, J. (2021). On-topic conversational responding in autistic and neuro-typical children. *European Society for Philosophy and Psychology Conference,* Leipzig, Germany. <https://doi.org/10.17605/OSF.IO/Q7WA4>

MacWhinney, B. (2000).  *The CHILDES Project: Tools for Analyzing Talk. Transcription format and programs* (3rd ed.).  Lawrence Erlbaum Associates.

Onea, E. (2013). *Potential Questions in Discourse and Grammar.* Habilitation Thesis, Universität Göttingen. <https://doi.org/10.13140/RG.2.1.1888.1128>

Onea, E. (2016). *Potential Questions at the Semantics-Pragmatics Interface*. BRILL. <https://doi.org/10.1163/9789004217935>

Pagmar, D., Abbot-Smith, K., & Matthews, D. (2022). Predictors of children’s conversational contingency. Language Development Research, 2(1), 139-179. <https://doi.org/10.34842/2022-511>

Riester, A. (2019). Constructing QUD trees. In *Questions in Discourse* (Vol. 2, pp. 164–193). BRILL. <https://doi.org/10.1163/9789004378322_007>

Roberts, C. (2012). Information structure in discourse: Towards an integrated formal theory of pragmatics. *Semantics and Pragmatics, 5,* 1–69. <https://doi.org/10.3765/sp.5.6>

Van Kuppevelt, J. (1995). Discourse structure, topicality and questioning. *Journal of Linguistics, 31,* 109-147. <https://doi.org/10.1017/S002222670000058X>

1. *Topic* is an elusive concept that has received different definitions in the literature. In the present work, we follow Van Kuppevelt (1995) and define topic in terms of *aboutness*, i.e., what the utterance is about. [↑](#footnote-ref-1)
